# One-Pot Amination of 5-Hydroxymethylfurfural to 2,5-Bis(aminomethyl)furan over NiZnAl Catalysts

**DOI:** 10.3390/molecules31101600

**Published:** 2026-05-10

**Authors:** Cong Wang, Xin Li, Junqi Zhao, Bin Sun, Xiaoxin Zhang, Xuhong Mu

**Affiliations:** State Key Laboratory of Petroleum Molecular & Process Engineering, SINOPEC Research Institute of Petroleum Processing, Beijing 100083, China

**Keywords:** 5-hydroxymethylfurfural, 2,5-bis(aminomethyl)furan, reductive amination, Ni-based catalyst, ZnAl_2_O_4_ spinel structure

## Abstract

This study developed a highly efficient one-step catalytic reductive amination method, achieving the highly selective conversion of 5-hydroxymethylfurfural (HMF) to 2,5-bis(aminomethyl)furan (BAMF). A series of NiZnAl catalysts were prepared via the coprecipitation method and demonstrated excellent catalytic performance in HMF conversion. The Ni_4_Zn_4_Al_8_O_x_ catalyst achieved up to 100% substrate conversion with 83.71% BAMF selectivity. Their structure–activity relationship was elucidated through a comprehensive characterization using XRD, H_2_-TPR, NH_3_-TPD, XPS, and TEM techniques. The study reveals that the unique synergistic interactions between the metal and acidic sites on the ZnAl_2_O_4_ spinel structure is crucial for catalytic performance: on the one hand, Zn introduction forms the spinel structure and promotes electron enrichment at Ni active sites, significantly enhancing the activation capability of HMF hydroxyl groups; on the other hand, the moderately acidic sites in the catalyst form “metal-acid” dual-functional synergistic centers with the metal sites, simultaneously promoting substrate activation and effectively regulating the transformation pathways of reaction intermediates. This precise matching between the metal active sites and acidic sites enables the efficient sequential progression of all steps in the reaction, offering a novel and more selective solution for the efficient reductive amination of HMF.

## 1. Introduction

5-Hydroxymethylfurfural (HMF) is a versatile C6 platform compound derived from renewable biomass feedstocks, including glucose, fructose, and cellulose [1,2]. As an important biobased platform compound, HMF exhibits broad application prospects and can be used to synthesize a variety of high-value-added monomers and fine chemicals [3]. Among its derivatives, 2,5-bis(aminomethyl)furan (BAMF) possesses a structurally stable furan ring skeleton. It is expected that it will replace traditional petroleum-based diamine monomers and be applied in polymer synthesis systems, serving as a promising monomer for the preparation of high-performance polymer materials. Meanwhile, it is further broadening the application scope of HMF in the field of biomass-derived materials. This work mainly summarizes and discusses the reaction pathway of the heterogeneous catalytic amination of HMF to BAMF (Table 1).

Diverse synthetic routes have been developed for the conversion of HMF to BAMF, among which the typical strategy involves the initial oxidation of HMF to 2,5-furandicarboxaldehyde (DFF), which is subsequently employed as the key precursor for BAMF synthesis. For instance, Le [4] utilized acetic acid-treated Raney Ni as the catalyst to facilitate the reductive amination of DFF, resulting in BAMF with a moderate yield of 42.6%. Indeed, the pronounced reactivity of DFF together with the intrinsic polymerization tendency of diamine products constitutes a major hurdle for the highly selective synthesis of BAMF. To address these issues, Qi [5] proposed a modified protocol by reacting DFF with a stoichiometric excess of n-butylamine, which enabled the formation of a sterically hindered imine intermediate that is less prone to condensation. By precisely modulating the hydrogenation activity of the Co/ZrO_2_ catalyst toward this intermediate, they successfully realized the highly selective transformation to BAMF, achieving an impressive yield of 95%. The selective hydrogenation of HMF produces 2,5-dihydroxymethylfuran (DHMF), upon which Kita [6] accomplished direct amination with ammonia over the heterogeneous catalyst Ru-20MgO/TiO_2_ at 110 °C, achieving a BAMF yield of 86%. Alternatively, replacing the hydroxyl groups in HMF with chlorine yields 5-chloromethylfurfural (5-CMF), which undergoes reductive amination with Raney Ni as the catalyst in THF at 90 °C, giving BAMF with a 62% yield [7,8]. In another synthetic strategy, Wang [9] first subjected HMF to a Ritter reaction to aminate the hydroxyl group, resulting in N-(hydroxymethylfurfuryl)acetamide (NAMF) with a yield of 89.1%, followed by the subsequent amination of NAMF over a Raney Ni catalyst to produce BAMF with a yield of 60.8%. Wei [10] developed a stepwise reduction amination protocol, wherein HMF was initially reacted with Raney Co at 120 °C for 2 h to preferentially aminate the C=O groups and generate the intermediate HMFA; the resultant HMFA was further treated with Raney Ni at 160 °C for 10 h to achieve the amination of the C-OH groups, ultimately yielding 88.3% BAMF. Although these multi-step strategies have enabled considerable progress and satisfactory product yields in the synthesis of target chemicals, the development of more efficient and convenient one-pot catalytic routes is still highly desirable for advancing practical applications.

Researchers have shifted from traditional multi-step procedures to an advanced one-pot catalytic system, which avoids intermediate separation and purification, greatly simplifies operation, and improves the atomic economy through in situ conversion. This transformation involves the simultaneous amination of both hydroxyl and aldehyde groups, which imposes stringent requirements on catalyst design and selection [11]. Zhou [12] systematically investigated the catalytic performance of Raney Ni, Raney Co, Pd/C, Ru/C, and Pt/C catalysts in the amination of HMF, and the results indicated that Raney Ni exhibited the highest catalytic efficiency. The DFT calculations further revealed that Ni possesses the minimal adsorption energy difference between NH_3_ and H_2_, which mitigates the competitive adsorption of NH_3_ on the metal active sites, thereby reserving more active centers for alcohol dehydrogenation and imine hydrogenation, and consequently enhancing the overall efficiency of the HMF amination reaction. Under the reaction conditions of 120 °C, 1 MPa H_2_ and 0.2 MPa NH_3_ for 2 h followed by elevating the temperature to 160 °C for an additional 10 h, a BAMF yield of 86.5% was attained. Additionally, Wei [13] prepared 10wt% Ni catalysts using *γ*-Al_2_O_3_, ZnO, TiO_2_, SiO_2_, activated carbon (AC) and hydrotalcite (HT) as supports via the incipient wetness impregnation method, and evaluated their catalytic behavior in the one-pot reductive amination of HMF. Among these supports, *γ*-Al_2_O_3_ demonstrated distinct advantages with a BAMF yield of 21.5%, whereas the other supports only enabled the amination of the C=O groups, leading exclusively to the formation of the intermediate HMFA. Although these pioneering studies have achieved encouraging progress in catalyst screening and performance optimization, the intrinsic reaction mechanism of HMF reductive amination remains ambiguous and requires further in-depth investigation.

Recent studies have further illustrated that the electronic state of Ni active sites is pivotal in regulating gas activation [14], intermediate transformation [15], and reaction pathway selection [16,17,18]. Meanwhile, Sels [3,19] has highlighted that the synergy between metal active sites and acidic sites plays a crucial role in controlling the functional group conversion and product selectivity during HMF catalytic transformation. A delicate balance between hydrogenation activity and acid-catalyzed side reactions is therefore essential for the rational design of efficient catalysts. Guided by these understandings, NiAl catalysts were initially prepared by a coprecipitation method. However, the excessively high hydrogenation activity of Ni led to side reactions, such as aldehyde group hydrogenation and furan ring hydrogenation. Meanwhile, the surface acidic sites induced side reactions, such as intramolecular cyclization, which not only decreased the yield of BAMF but also increased the difficulty of its subsequent separation and purification. To solve this problem, this work innovatively introduces Zn into NiAl catalysts to form a ZnAl_2_O_4_ spinel structure. The spinel structure eliminates the strong acid sites responsible for the cyclization side reaction and establishes strong metal–support interactions, thereby modulating the electronic structure of Ni and optimizing its hydrogenation activity. By adjusting the Ni/Zn/Al molar ratio, this study demonstrates how this dual regulation realizes the reductive amination of HMF to BAMF. This design strategy, which combines the electronic structure regulation of Ni active sites and the acid–base property optimization of the support, provides a new approach for the design of bifunctional catalysts in biomass conversion.

**Table 1:** Summary and comparison of heterogeneous catalysts for the synthesis of BAMF from HMF and its derivatives.

Reaction Pathway	Catalyst	Reaction Conditions	BAMF Yield (%)	Ref.
DFF-BAMF	Raney Ni	120 °C, 6 h, 1 MPa	42.6	[4]
DFF-BAMF	Co/ZrO_2_	100 °C, 10 h, 2 MPa	95.0	[5]
DHMF-BAMF	Ru-20MgO/TiO_2_	110 °C, 20 h, 0.7 MPa	86.0	[6]
CMF-BAMF	Renay Ni	90 °C, 1 h, 4 MPa	62.0	[8]
NAMF-BAMF	Renay Ni	120 °C, 3 h, 1.5 MPa	60.8	[9]
HMFA-BAMF	Raney Ni	160 °C, 48 h, 1 MPa	71.1	[10]
HMF-BAMF	Raney Ni	120 °C, 2 h and then 160 °C, 10 h, 1 MPa	86.5	[12]
HMF-BAMF	10Ni/*γ*-Al_2_O_3_	160 °C, 36 h, 1 MPa	86.3	[13]
This work	Ni_4_Zn_4_Al_8_O*_x_*	90 °C for 6 h and then 210 °C, 18 h, 4.5 MPa	83.7	-

## 2. Results and Discussion

### 2.1. Catalyst Characterization

**Figure 1:**
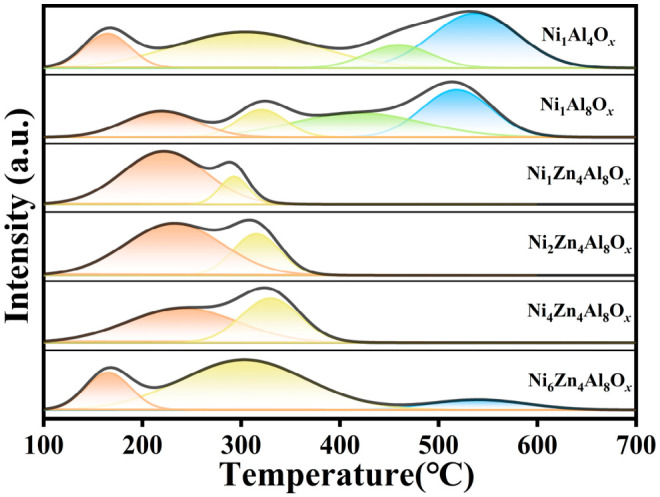
NH_3_-TPD of the prepared catalysts.

The NH_3_-TPD characterization and acid amount quantification revealed distinct differences in the number, strength, and distribution of acidic sites across the catalysts (Figure 1). The total acid amounts of Ni_1_Al_4_O*_x_* and Ni_1_Al_8_O*_x_* were 0.067 μmol/g and 0.061 μmol/g, respectively, which were significantly higher than those of the NiZnAl catalysts. Notably, the strong acid sites (400–600 °C) account for about 40–45% of the total acid amount in the NiAl catalysts, and these excessively strong acidic centers are prone to triggering acid-catalyzed cyclization side reactions, leading to the formation of OABCO byproducts and severely compromising the selectivity toward BAMF [20].

Upon Zn incorporation, the high-temperature desorption peaks associated with the strong acid sites vanished completely in the NiZnAl catalysts. The acidic sites were predominantly confined to the medium-to-low temperature region (150–400 °C), with the total acid amount drastically reduced to 0.032–0.045 μmol/g. Specifically, the moderate acid sites (250–400 °C) became the dominant species, accounting for about 30–70% of the total acid amount. This result confirms that Zn effectively suppresses the formation of strong acid sites and neutralizes part of the acidic centers, thus constructing a moderate acid site system on NiZnAl catalysts and tailoring the acid strength distribution to match the metal active sites. Notably, the reduced total acid amount and suppressed strong acid sites effectively inhibited acid-catalyzed side reactions. The cleavage product FA was eliminated completely, and the cyclization byproduct OABCO was drastically reduced, which contributed significantly to the improved selectivity toward BAMF.

The textural properties of various catalyst series were systematically characterized using N_2_ adsorption–desorption isotherms (Figure S1), pore size distribution analysis, and mesostructural parameters (Table 2). All samples displayed Type IV isotherms, which are typical of mesoporous materials, as evidenced by the sharp adsorption uptakes at high relative pressures, where the *P/P*_0_ was greater than 0.8. Notably, the Ni_1_Al_4_O*_x_* and Ni_1_Al_8_O*_x_* catalysts exhibited high specific surface areas of approximately 200 m^2^/g with narrow pore size distributions ranging from 6.91 to 7.73 nm, as confirmed by the pronounced peaks in their pore size distribution curves. In stark contrast, the NiZn catalysts demonstrated markedly reduced BET surface areas of 10 to 12 m^2^/g alongside enlarged pore sizes and diminished pore volumes, indicative of a poorly developed, loose pore architecture. The NiZnAl catalysts exhibited intermediate textural properties, bridging the two extremes. Their pore size distribution curves showed attenuated peak intensities relative to the Ni_1_Al_8_O*_x_* catalyst, accompanied by broader pore size ranges. As the pore size increased, the dV/dlogD profiles progressively flattened, suggesting greater pore heterogeneity and a higher fraction of larger mesopores.

Interestingly, the NiZnAl catalysts displayed composition-dependent adsorption behavior: the S_BET_ initially rose with an increasing Ni content before plateauing, while the average pore diameters slightly contracted. These findings demonstrate that precise control over the Ni/Zn/Al molar ratio enables rational modulation of pore structure development, including the total pore volume and pore size distribution characteristics.

**Figure 2:**
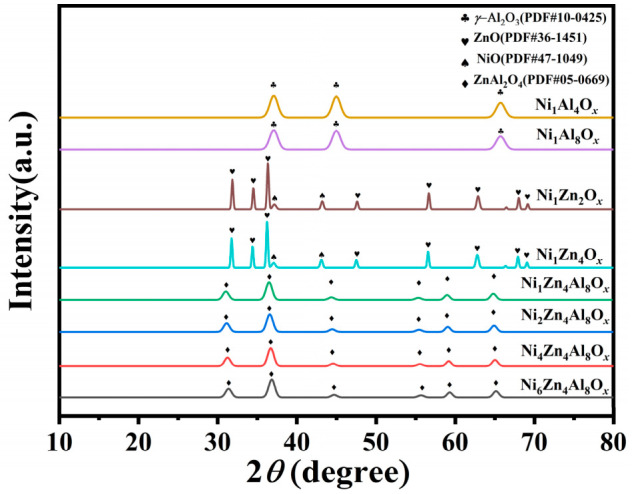
XRD of the NiZnAl catalysts.

The XRD diffraction patterns are shown in Figure 2. For the NiAl catalysts, the characteristic diffraction peaks assigned to *γ*-Al_2_O_3_ (PDF#10-0425) were only observed at 2*θ* values of approximately 37.5°, 45.8°, and 66.8°, corresponding to the (311), (400), and (440) crystal planes, respectively. No distinct peaks related to NiO were detected in the patterns, revealing that the Ni species were highly dispersed within the *γ*-Al_2_O_3_ support or dissolved into the *γ*-Al_2_O_3_ lattice to form a solid solution. For the NiZn catalysts, several sharp and well-defined diffraction peaks, ascribed to ZnO (PDF#36-1451) and NiO (PDF#47-1049), were present. Concretely, the diffraction peaks at 31.8°, 34.4°, 36.3°, 47.5°, 56.6°, 62.9°, and 67.9° correspond to the typical crystal planes of ZnO, while the peaks located at 37.2° and 43.3° are attributed to the (111) and (200) planes of NiO, respectively. This result confirms the coexistence of crystalline ZnO and NiO phases in the NiZn catalysts, and that no spinel phase was formed in the absence of Al. New characteristic diffraction peaks ascribed to the ZnAl_2_O_4_ spinel phase (PDF#05-0669) were detected in the NiZnAl catalysts. These peaks were located at 2*θ* values of about 31.3°, 36.7°, 44.7°, 55.5°, 59.2° and 65.0°, which were well indexed to the (220), (311), (400), (422) and (440) crystal planes of ZnAl_2_O_4_ spinel phase. This result proves that the ZnAl_2_O_4_ spinel structure was successfully synthesized. Notably, compared with the NiZn catalysts, the characteristic diffraction peaks of NiO vanished completely in all the NiZnAl catalysts. This phenomenon suggests that the Ni^2+^ ions were incorporated into the ZnAl_2_O_4_ spinel lattice to form a NiZnAl spinel solid solution, or were highly dispersed on the spinel surface via strong metal–support interactions [21,22].

**Figure 3:**
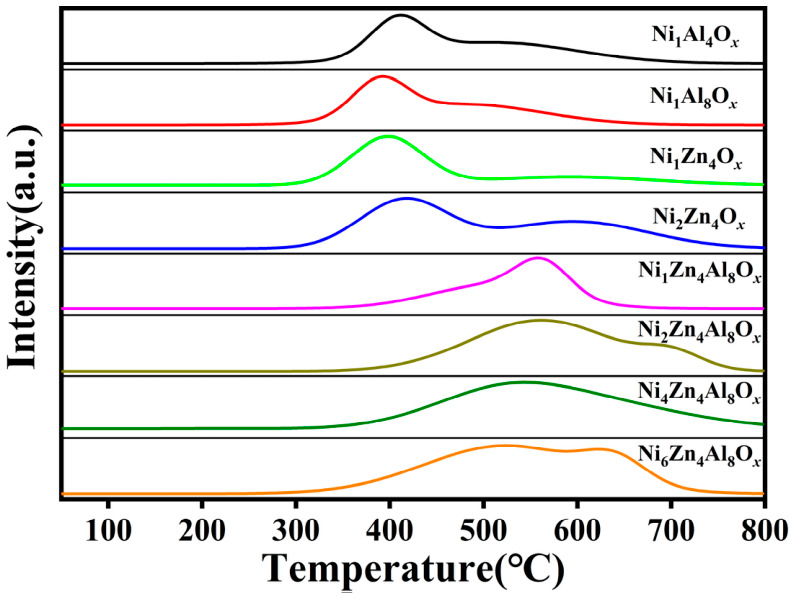
H_2_-TPR of the NiZnAl catalysts.

Hydrogen temperature-programmed reduction (H_2_-TPR) tests were conducted on the calcined catalysts to evaluate their reductive properties (Figure 3). The reduction peaks of the Ni_1_Al_4_O*_x_*, Ni_1_Al_8_O*_x_*, Ni_1_Zn_4_O*_x_* and Ni_2_Zn_4_O*_x_* catalysts were concentrated in the temperature range of 350–450 °C, with relatively low peak temperatures, implying that the Ni species in these catalysts possessed facile reducibility. In sharp contrast, the reduction peak temperatures of the NiZnAl catalysts were markedly higher than those of the aforementioned two catalyst categories. This phenomenon can be ascribed to the strong interfacial interactions formed between the Ni species and the ZnAl_2_O_4_ spinel structure introduced into the NiZnAl catalysts, which increased the reduction difficulty of Ni^2+^ species in the system, and thus led to a distinct shift of the reduction peaks toward the high-temperature region [23]. Notably, this observation not only further confirms the formation of the ZnAl_2_O_4_ spinel structure but also demonstrates that the influence of such interfacial interactions becomes increasingly pronounced with an increase in the Ni content.

The H_2_-TPD (Table S2) reveals distinct hydrogen adsorption–desorption behaviors among the catalysts. The Ni_1_Al_4_O*_x_* and Ni_1_Al_8_O*_x_* catalysts exhibited prominent H_2_ desorption peaks in the medium–high temperature range (400–600 °C), with corresponding hydrogen adsorption capacities of 8.2 mmol/g and 7.8 mmol/g, respectively, which were significantly higher values than those observed for the NiZnAl catalysts. This demonstrates the presence of strong hydrogen adsorption sites on the NiAl catalysts, which enhanced hydrogen dissociation and resulted in high hydrogenation activity. Notably, these characteristics correlate with the higher yield of furan ring hydrogenation byproducts observed with the NiAl catalysts. Among the NiZnAl catalysts, Ni_1_Zn_4_Al_8_O*_x_* exhibited the lowest H_2_ adsorption capacity at 4.3 mmol/g, followed by Ni_2_Zn_4_Al_8_O*_x_* at 5.1 mmol/g, Ni_4_Zn_4_Al_8_O*_x_* at 5.6 mmol/g, and Ni_6_Zn_4_Al_8_O*_x_* at 6.5 mmol/g. Among these, the Ni_4_Zn_4_Al_8_O*_x_* catalyst demonstrated a moderate H_2_ adsorption capacity, a key feature of Ni active sites on the ZnAl_2_O_4_ spinel support for matching the moderate acid sites. This balanced H_2_ adsorption capacity provided the Ni with appropriate hydrogenation activity. This allowed it to form a highly efficient metal–acid synergistic system with the moderate acid sites on the ZnAl_2_O_4_ spinel support. This synergy not only facilitated the selective conversion of HMFA to BAMF, but also prevented both excessive hydrogenation of the furan ring and the subsequent intramolecular cyclization side reactions. In contrast, the lower H_2_ adsorption capacity of the Ni_1_Zn_4_Al_8_O*_x_* and Ni_2_Zn_4_Al_8_O*_x_* catalysts, ranging from 4.3 to 5.1 mmol/g, resulted in insufficient hydrogenation activity, limiting HMFA conversion and leading to low BAMF selectivity. The Ni_6_Zn_4_Al_8_O*_x_* catalyst exhibited an excessive H_2_ adsorption capacity, with the Ni demonstrating overly high hydrogenation activity. This disrupted the balanced synergy between the metal–acid sites on the ZnAl_2_O_4_ spinel support, leading to the uncontrolled hydrogenation of the furan ring.

**Figure 4:**
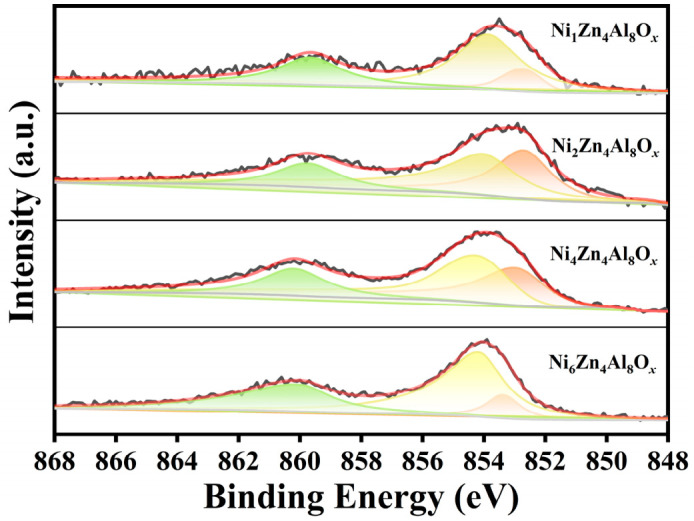
XPS spectra of Ni 2p_3/2_ core level for the NiZnAl catalysts.

X-ray photoelectron spectroscopy (XPS) was employed to investigate the chemical states of elements on the catalyst surfaces. The high-resolution Ni 2p_3/2_ spectrum of the calcined catalyst is shown in Figure 4 and Table S2. The Ni 2p_3/2_ binding energy of NiZnAl catalysts gradually increased from 852.70 eV (Ni_1_Zn_4_Al_8_O*_x_*), to 852.75 eV (Ni_2_Zn_4_Al_8_O*_x_*), to 852.98 eV (Ni_4_Zn_4_Al_8_O*_x_*), and ultimately to 853.36 eV (Ni_6_Zn_4_Al_8_O*_x_*) with a rising Ni content. This result indicates that increasing the Ni loading progressively strengthened the metal–support electronic interactions between the Ni species and the ZnAl_2_O_4_ spinel structure, thereby modifying the electronic structure of Ni active sites. At low Ni loadings (Ni_1_Zn_4_Al_8_O*_x_* Ni_2_Zn_4_Al_8_O*_x_*), the relatively low Ni 2p_3/2_ binding energy (852.70–852.75 eV) indicates weak electronic modulation from the spinel structure [24]. Correspondingly, the H_2_-TPD reveals a low H_2_ adsorption capacity, which in turn limited the efficiency of H_2_ adsorption capacity and dissociation on the catalyst, hindering the rate-limiting HMFA dehydrogenation step, thus resulting in low BAMF selectivity. For the Ni_4_Zn_4_Al_8_O*_x_* catalyst (Ni 2p_3/2_ = 852.98 eV), the moderate binding energy further confirms a balanced metal–support interaction on the ZnAl_2_O_4_ spinel support, which realized the precise matching of electronic structure of Ni active sites and the acidity of surface acid sites. This electronic tuning weakened the excessive H_2_ adsorption capacity, matching the intermediate H_2_ adsorption capacity observed in the H_2_-TPD. The resulting equilibrium of H_2_ adsorption–dissociation favored imine double-bond reduction over furan ring saturation, enabling efficient HMFA amination to BAMF. In contrast, the Ni_6_Zn_4_Al_8_O*_x_* catalyst exhibited the highest Ni 2p_3/2_ binding energy among all the NiZnAl samples, reaching 853.36 eV. This indicates a disruption of the favorable electronic regulation of the ZnAl_2_O_4_ spinel structure on the Ni active sites. The corresponding H_2_-TPD results reveal a higher H_2_ adsorption capacity, yet the Ni sites displayed an uncontrolled H_2_ adsorption capacity and dissociation. This broke the synergistic balance between the Ni metal and acidic sites. As a result, excessive hydrogenation of the furan ring occurred, thus forming HMTHFA and DHMTHF.

**Figure 5:**
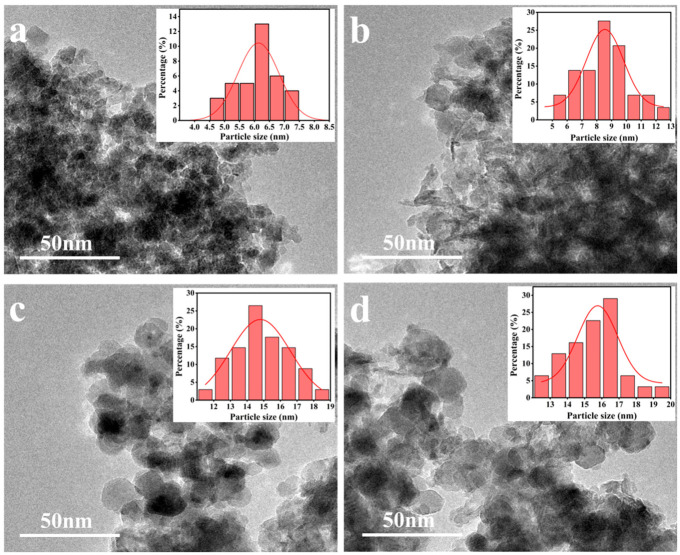
TEM images of the (**a**) Ni_1_Zn_4_Al_8_O*_x_*, (**b**) Ni_2_Zn_4_Al_8_O*_x_*, (**c**) Ni_4_Zn_4_Al_8_O*_x_*, and (**d**) Ni_6_Zn_4_Al_8_O*_x_* catalysts.

The TEM analysis coupled with the particle size distribution measurements of the NiZnAl catalysts provides compelling evidence regarding the Ni loading-dependent evolution of metallic Ni nanoparticles (Figure 5). At lower Ni contents (Ni_1_Zn_4_Al_8_O*_x_*, Ni_2_Zn_4_Al_8_O*_x_*, and Ni_4_Zn_4_Al_8_O*_x_* catalysts), highly dispersed Ni nanoparticles with narrow size distributions were observed: the average particle sizes derived from the TEM were 6.5 nm, 8.8 nm, and 14.6 nm, respectively. However, increasing the Ni contents induced morphological alterations. For the Ni_6_Zn_4_Al_8_O*_x_* catalyst, the Ni particles were significantly enlarged, with an average particle size of 16.2 nm, accompanied by conspicuous agglomeration phenomena as verified by the broader particle size histograms. This deteriorated dispersion exposed residual strong acidic sites, which accounts for the observed enhancement in cyclization side reactions and the consequent decline in BAMF selectivity. A possible reason for the inferior catalytic performance is that excessive Ni loading may compromise dispersion and undesirably expose catalytically detrimental acidic sites. Nevertheless, it should be emphasized that the Ni particle size itself may also exert an independent intrinsic effect on catalytic activity, which still warrants further in-depth investigation in future research.

### 2.2. Catalyst Performance Results

This study finds that during the preparation of BAMF from HMF, the aldehyde group in the HMF molecules exhibits high chemical reactivity and readily undergoes multiple competing reactions. Therefore, it is essential to prioritize the reaction between the aldehyde group and ammonia, followed by hydrogenation to obtain the target intermediate HMFA. Two primary reaction pathways exist: one involves HMF dehydrating with NH_3_ to form an imine intermediate, which is then hydrogenated to yield HMFA and subsequently converted to BAMF; the other involves HMF condensing with the generated HMFA to form a Schiff base intermediate through dehydration, which is then hydrogenated to produce BAMF. The imine intermediate pathway is more sensitive to temperature changes, remaining stable at 90 °C but rapidly converting when the temperature rises to 120 °C.

Table S1 indicates that without a catalyst, at 90 °C, the imine and Schiff base intermediates initially increased and then decreased over time. HMFA started to form at 6 h, with only a small amount of BAMF appearing at 10 h. At 120 °C, the imine intermediate completely disappeared, the Schiff base intermediate rapidly decreased, and HMFA reached a 92.52% yield at 10 h, with no BAMF formation. Over the Ni_4_Zn_4_Al_8_O*_x_* catalyst at 90 °C, the complete conversion of imine and Schiff base intermediates occurred within 6 h, yielding 92.89% HMFA with a small amount of BAMF (3.84%); at 120 °C, the catalyst accelerated the conversion of HMFA to BAMF, resulting in a reduced HMFA yield and increased formation of byproducts. This comprehensive analysis indicates that to ensure the active aldehyde group in HMF molecules is preferentially converted to an amine group, the reaction condition of 90 °C for 6 h is necessary to maximize the conversion of intermediates to HMFA.

The Ni_1_Al_4_O*_x_* catalyst exhibited undesired product distribution characteristics, yielding merely 41.72% selectivity for BAMF (Table 3, entry 1). Instead, significant formation of byproducts was observed, including furfurylamine (FAM 2.05%), 5-methyl-2-furanamine (MFAM 2.08%), 2,5-dihydroxymethylfuran (DHMF 5.47%), 2,5-dihydroxymethyltetrahydrofuran (DHMTHF 9.50%), and the cyclization product 8-oxa-3-azabicyclo [3.2.1]octane (OABCO 4.26%). It is worth noting that with a further increase in the Al content of the Ni_1_Al_8_O*_x_* catalyst (Table 3, entry 2), the selectivity for BAMF further decreased to 28.56%, accompanied by significant HMFA accumulation. Meanwhile, the types of byproducts were similar to those of the Ni_1_Al_4_O*_x_* catalyst (including FAM, MFAM, DHMF, DHMTHF, OABCO, etc.), with only slight variations in their proportions. These results indicate that the catalytic system suffered from excessive hydrogenation activity, promoting undesirable side reactions, such as over-hydrogenation, intramolecular cyclization, and C-C bond scission. In contrast, although the Ni_1_Zn_2_O*_x_* and Ni_1_Zn_4_O*_x_* catalysts demonstrated low BAMF selectivity, they exhibited superior selectivity toward total amines (BAMF + HMFA) with minimal side-product formation (Table 3, entries 3 and 4). This suggests that NiZn catalysts suppress competitive side reactions but lack the ability to efficiently promote the critical secondary amination step for converting HMFA to BAMF.

For the Ni_1_Zn_4_Al_8_O*_x_* catalyst, a significant improvement was observed compared to the pure NiZn catalysts, demonstrating enhanced conversion of HMFA to BAMF, with the total amines (BAMF + HMFA) selectivity reaching 94.68% (Table 3, entry 5). When the Ni/Zn/Al molar ratio was increased from 1:4:8 to 2:4:8 and 4:4:8, the conversion of HMFA to BAMF was significantly improved. The Ni_4_Zn_4_Al_8_O_x_ catalyst demonstrated the best performance, achieving a BAMF selectivity of 83.71% while maintaining a total amines selectivity consistently above 90% (Table 3, entry 7). After being reused five times, the catalyst showed no significant loss in activity, and its spinel structure was well maintained without obvious structural alteration (Figure S2). Notably, its byproduct formation was substantially lower than that with the NiAl catalysts, highlighting the NiZnAl catalysts’ dual advantages of maintaining high main product selectivity while effectively suppressing various side reactions, thus realizing a concentrated product distribution. However, further increasing the Ni content to Ni/Zn/Al = 6:4:8 resulted in a decline in the amination performance of the Ni_6_Zn_4_Al_8_O*_x_* catalyst, with the BAMF selectivity decreasing to 73.68% and the total amines selectivity dropping to 78.96% (Table 3, entry 8).

### 2.3. Study of the Reaction Mechanism

**Figure 6:**
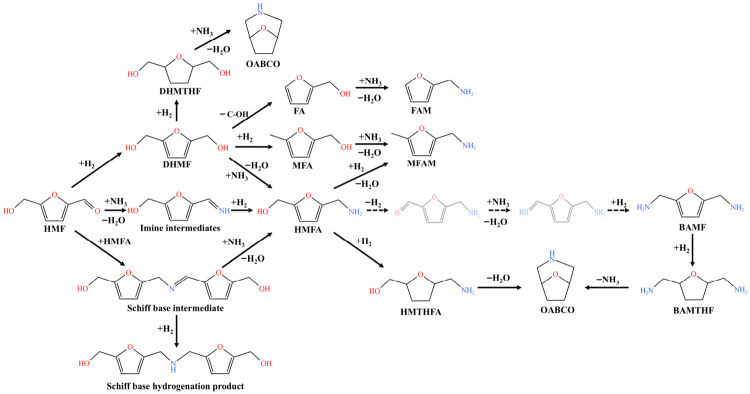
The pathways for the formation of BAMF and byproducts in the reductive amination of HMF.

For the HMF reduction amination reaction, the various products were identified by GC-MS (Figures S3–S16). Based on the distribution of the products, possible reaction pathways were proposed. The one-step reductive amination reaction mechanism of HMF and the byproduct formation pathway are illustrated in Figure 6. The reaction mechanism for HMF conversion to BAMF comprises two core steps: (1) Nucleophilic addition of NH_3_ to the C=O double bond in HMF molecules generates an imine intermediate. The Ni metal sites dissociate H_2_ into reactive hydrogen species (H*) [12], reduce the C=N double bond of the imine intermediate to a saturated -NH_2_ group, and generate HMFA. Alternatively, HMF can also form HMFA via a Schiff base process [25,26,27]. (2) The Ni metal sites catalyze the dehydrogenation of the hydroxymethyl group in HMFA, generating an aldehyde-containing intermediate [28,29]; the reactivated aldehyde intermediate reacts with NH_3_ to form a new imine intermediate, and the Ni sites catalyze the hydrogenation of the C=N double bond to a saturated C-N bond, ultimately yielding the target product BAMF [20].

The byproduct formation mechanisms in this system can be described as follows. In the NiAl catalysts, the highly active Ni sites catalyze the hydrogenation of aldehyde group within the HMF to form DHMF. The DHMF then undergoes chain cleavage and hydrogenation–deoxygenation to yield FA and MFA, respectively. These two products further undergo amination to form FAM and MFAM, respectively. The highly active Ni sites are also capable of catalyzing the hydrogenation of C=C double bond in the furan ring of HMFA/BAMF/DHMF molecules, thereby forming HMTHFA/BAMTHF/DHMTHF [30]. Under the synergy of acidic sites, these intermediates undergo intramolecular cyclization, releasing one molecule of H_2_O or one molecule of NH_3_ to form OABCO [31,32]. The ZnAl_2_O_4_ spinel structure suppresses both of the above pathways simultaneously. Specifically, the strong metal–support interaction induces a downshift of the d-band center of Ni, which weakens the π interaction between the Ni sites and the furan ring. This not only ensures the dehydrogenation of hydroxymethyl groups in HMFA, but also suppresses excessive hydrogenation side reactions. Furthermore, the cyclization reactions are inhibited by eliminating the strong acidic sites.

## 3. Experiment

### 3.1. Chemicals

HMF (97%), HMFA (99%), BAMF (98%), FA (99%), FAM (99%), MFA (99%), MFAM (99%), DHMF (99%), BATHMF (97%), OABCO (98%), 1,4-Dioxane (AR), Ni(NO_3_)_2_·6H_2_O (AR), Al(NO_3_)_3_·9H_2_O (AR), Zn(NO_3_)_2_·6H_2_O (AR), Na_2_CO_3_ (AR), and NaOH (AR) were used.

All chemical reagents were purchased from Beijing InnoChem Science & Technology Co., Ltd. (Beijing, China) and used directly without any further purification.

### 3.2. Preparation of the Catalysts

The NiZnAl catalyst was synthesized using the coprecipitation method. Appropriate amounts of Ni(NO_3_)_2_·6H_2_O, Zn(NO_3_)_2_·6H_2_O, and Al(NO_3_)_3_·9H_2_O were weighed and dissolved in deionized water to obtain a mixed solution. A mixed aqueous solution of 1.0 mol/L Na_2_CO_3_ and 1.0 mol/L NaOH was used as the precipitating agent. The mixed solution and precipitating agent were added dropwise in a co-current manner into a beaker, with their flow rates adjusted to maintain the pH between 9 and 10 during precipitation. After completion of the addition, the mixture was stirred at 50 °C for 3 h. The catalyst precursor was then aged at ambient temperature for 12 h. Following aging, the sample was washed repeatedly with deionized water until the filtrate pH reached 7. The washed catalyst was dried at 120 °C for 20 h, followed by calcination at 600 °C in a muffle furnace for 3 h. The prepared catalyst was ground after calcination and placed in a tube furnace for reduction. Controlling the hydrogen flow rate at 100 mL/min, the reduction was conducted at 550 °C for 3 h. After cooling to room temperature, it was passivated with 2% O_2_ concentration inert gas for 0.5 h. The resulting catalyst was designated as the Ni_a_Zn_b_Al_c_O*_x_* catalyst. The NiAl and NiZn catalysts were also synthesized via the same coprecipitation procedure. The NiAl catalyst was prepared without the addition of Zn(NO_3_)_2_·6H_2_O, while the NiZn catalyst was synthesized in the absence of Al(NO_3_)_3_·9H_2_O. The subsequent steps, including co-current precipitation, aging, washing, drying, calcination, reduction, and passivation, were identical to those described above for the NiZnAl catalysts. The obtained catalysts were denoted as Ni_a_Al_c_O*_x_* and Ni_a_Zn_b_O*_x_*, (a, b, and c represent the molar ratios of Ni, Zn, and Al, respectively).

### 3.3. Methods for Catalyst Characterization

XRD patterns of the samples were collected on an Empyrean X-ray diffractometer equipped with filtered Cu Kα radiation; the measurements were conducted at a tube current of 40 mA and a tube voltage of 40 kV, with a scanning rate of 10°/min over a 2θ range of 10°–80°.

XPS analysis of the prepared catalysts was performed using an ESCA Lab 250 X-ray photoelectron spectrometer with Al Kα radiation as the excitation source in dual-anode mode. First, a survey spectrum was recorded over the binding energy range of 0–1200 eV with an energy step of 0.5 eV. Subsequently, the individual elemental spectra were recorded with an energy step of 0.05 eV, including C (275–305 eV), O (520–540 eV), and Ni (835–900 eV). The peak shifts in all spectra were corrected using the intrinsic carbon peak at 284.8 eV.

The XRF analysis was carried out on a ZSX-2 X-ray fluorescence spectrometer. The instrument was operated at an excitation voltage of 50 kV and an excitation current of 50 mA using an Rh target. The elemental composition of the catalyst sample was determined based on the characteristic X-ray intensities of the detected elements, combined with standard calibration and theoretical calculation.

The TEM was conducted on a Philips (Amsterdam, The Netherlands) Tecnai F30 microscope operated at an accelerating voltage of 300 kV. The sample was ground into fine powder, dispersed in anhydrous ethanol, and ultrasonicated for 0.5 h to achieve uniform dispersion. The supernatant was dropped onto a carbon-coated copper grid and dried naturally under ambient conditions before TEM observation.

The H_2_ temperature-programmed reduction (H_2_-TPR) experiments were conducted on a Micromeritics (Norcross, GA, USA) AutoChem II 2920 chemisorption analyzer. Specifically, the catalyst samples were first pretreated at 150 °C for 1 h under a flowing argon atmosphere with a flow rate of 40 mL/min. After cooling the pretreated samples to ambient temperature, the TPR test was initiated by heating the samples to 800 °C at a ramp rate of 10 °C/min under a 10% H_2_/Ar mixed gas flow (30 mL/min), with the reduction signals recorded using a thermal conductivity detector (TCD).

The NH_3_ temperature-programmed desorption (NH_3_-TPD) experiments were performed as follows: After loading the catalyst sample into the reactor, the sample was first reduced at 400 °C for 10 h under a hydrogen atmosphere. Subsequently, the gas was switched to helium for a 10 min purge to remove the residual hydrogen. The sample was then cooled down to 100 °C, followed by purging with a 10%NH_3_/90%He mixed gas for 30 min until the NH_3_ adsorption on the sample surface reached saturation. After that, the gas was switched back to helium and purged continuously until the detector baseline stabilized. Finally, the sample was heated to 700 °C at a ramp rate of 10 °C/min under a continuous helium flow and maintained at this temperature; the desorption signals were recorded simultaneously by both the TPD detector and the mass spectrometer for the tail gas.

The GC-MS analyses were performed on a Thermo Scientific (Waltham, MA, USA) system consisting of a TRACE (Sydney, Australia) 1300 GC instrument with an ISQ 7000 mass spectrometer (Waltham, MA, USA) and a flame ionization detector (FID). Separation was achieved using a DB-17 capillary column (60 m × 0.25 mm × 0.25 μm). Helium was used as the carrier gas. The samples were injected in split mode at an inlet temperature of 250 °C, with a split ratio of 25:1 and a split flow rate of 50.0 mL/min. The oven temperature program was as follows: an initial temperature of 80 °C held for 15 min, then ramped at 10 °C/min to 300 °C and held for 5 min, resulting in a total run time of 42 min. For MS detection, the transfer line and ion source temperatures were maintained at 300 °C, and the electron ionization (EI) mode was employed. The FID detector was operated at 300 °C, with the gas flows set to 350 mL/min for air, 35 mL/min for hydrogen, and 40 mL/min for the makeup gas.

### 3.4. Catalytic Experiments

The reductive amination of HMF was carried out in a 100 mL stainless-steel high-pressure batch reactor under a stirring rate of 1000 rpm. Specifically, the reactor was sequentially charged with 5 g of HMF, 0.4575 g of Na_2_CO_3_, 25 mL of dioxane, and 1 g of the prepared catalyst. Prior to the reaction initiation, the air inside the reactor was purged five times with nitrogen gas, followed by three additional purges with high-purity hydrogen to ensure the complete elimination of residual oxygen and air. Subsequently, 11.5 g of ammonia gas was introduced into the reactor, and hydrogen gas was further supplemented to adjust the total initial pressure of the reaction system to 4.5 MPa. The reaction was conducted in a stepwise temperature-programmed manner: first incubated at 90 °C for 6 h, and then the temperature was elevated to 210 °C for 18 h. Upon the completion of the reaction, the reactor was naturally cooled to ambient temperature; the liquid product mixture was collected, subjected to vacuum filtration to remove the solid catalyst, and finally analyzed by gas chromatography–mass spectrometry (GC-MS, Thermo-ISQ 7000, Thermo Scientific, Waltham, MA, USA). Calculation of product composition: The conversion of HMF and product selectivity were calculated using the area normalization method.

## 4. Conclusions

This work addresses a core dilemma encountered in the reductive amination of HMF to BAMF, which involves balancing the sufficient hydrogenation activity required for HMF conversion with the suppression of side reactions, such as excessive hydrogenation and acid-catalyzed cyclization. NiZnAl catalysts were fabricated via the coprecipitation method by introducing Zn into a NiAl system to form a ZnAl_2_O_4_ spinel structure, achieving the synergistic optimization of Ni active sites and surface acidic sites. The ZnAl_2_O_4_ spinel structure not only removed the strong acidic sites and reduced the total acid content on the catalyst surface, but also generated a strong metal–support interaction, thereby modifying the electronic structure of Ni and tuning its hydrogenation activity to an appropriate level. The optimized Ni_4_Zn_4_Al_8_O*_x_* catalyst exhibited a BAMF selectivity reaching 83.71%. This study preliminarily clarifies the structure–activity relationship of the NiZnAl catalysts and proposes a possible reaction mechanism for the reductive amination of HMF, providing a vital theoretical basis for the design of high-performance catalysts for BAMF production.

## Figures and Tables

**Table 2:** Physical properties of the prepared catalysts.

Catalyst	Ni (wt.%)	Zn (wt.%)	Al (wt.%)	S_BET_ (m^2^/g)	Pore Size (nm)	Pore Volume (cm^3^/g)
Ni_1_Al_4_O*_x_*	12.1	—	44.7	199.79	7.73	0.41
Ni_1_Al_8_O*_x_*	20.5	—	37.8	204.94	6.91	0.39
Ni_1_Zn_4_O*_x_*	14.7	65.4	—	10.69	13.61	0.04
Ni_2_Zn_4_O*_x_*	24.7	55.1	—	11.59	23.87	0.07
Ni_1_Zn_4_Al_8_O*_x_*	7.3	32.3	26.7	110.02	9.03	0.25
Ni_2_Zn_4_Al_8_O*_x_*	13.3	29.6	24.4	131.17	13.14	0.38
Ni_4_Zn_4_Al_8_O*_x_*	23.5	24.3	19.6	167.60	8.04	0.35
Ni_6_Zn_4_Al_8_O*_x_*	29.8	22.1	18.3	165.47	8.23	0.32

**Table 3:** Reductive amination of HMF over different catalysts.

Catalyst	Conversion of HMF/%	Selectivity/%													Mass Balance/%
		BAMF	HMFA	BAMF + HMFA	FAM	FA	MFAM	MFA	OABCO	DHMF	BATHMF	HMTHFA	DHTHMF	Others	
Ni_1_Al_4_O*_x_*	100	41.72	28.90	70.62	2.05	0.79	2.08	-	4.26	5.47	0.53	2.23	9.50	2.47	97.06
Ni_1_Al_8_O*_x_*	100	28.56	48.24	76.80	0.80	0.44	0.60	-	2.20	5.56	0.38	2.64	6.79	3.79	96.00
Ni_1_Zn_2_O*_x_*	100	28.40	68.63	97.03	0.26	-	0.60	0.22	0.37	-	0.06	0.11	0.62	0.73	99.23
Ni_1_Zn_4_O*_x_*	100	41.58	50.06	91.64	0.67	-	0.99	0.19	0.78	-	0.12	0.35	1.91	3.35	96.54
Ni_1_Zn_4_Al_8_O*_x_*	100	49.92	44.76	94.68	0.26	-	0.81	-	0.89	-	0.25	1.52	1.27	0.32	99.64
Ni_2_Zn_4_Al_8_O*_x_*	100	76.52	13.49	90.01	0.92	-	-	-	0.65	-	0.37	1.61	1.41	5.03	94.82
Ni_4_Zn_4_Al_8_O*_x_*	100	83.71	8.33	92.04	-	-	-	-	-	-	0.41	1.32	1.37	4.86	95.14
Ni_6_Zn_4_Al_8_O*_x_*	100	73.68	5.28	78.96	-	-	0.74	-	2.47	1.24	0.48	2.67	9.42	4.02	95.98

Reaction conditions: 1 g catalyst; 5 g HMF; 25 mL 1,4-Diox; 11.5 g NH_3_; 4.5 MPa H_2_; 90 °C for 6 h and then 210 °C for 18 h.

## Data Availability

The original contributions presented in this study are included in the article/Supplementary Materials. Further inquiries can be directed to the corresponding authors.

## Supplementary Materials

The following supporting information can be downloaded at: https://www.mdpi.com/article/10.3390/molecules31101600/s1. Table S1: Reductive amination of HMF over Ni_4_Zn_4_Al_8_O_x_ catalyst; Figure S1: (**a**) N_2_ adsorption–desorption isotherms and (**b**) Pore size distribution diagram of the prepared catalysts; Table S2: Chemical property of the NiZnAl catalysts; Figure S2: (**a**) Effect of recycling times on the catalytic performance of the Ni_4_Zn_4_Al_8_O_x_ catalyst; (**b**) XRD patterns of fresh and 5 times recycled Ni_4_Zn_4_Al_8_O_x_ catalyst; (**c**) Ni 2p3/2 XPS spectra of fresh and 5 times recycled Ni_4_Zn_4_Al_8_O_x_ catalyst; (**d**) Zn 2p3/2 XPS spectra of fresh and 5 times recycled Ni_4_Zn_4_Al_8_O_x_ catalyst; Figure S3: Mass spectrum of HMFA; Figure S4: Mass spectrum of BAMF; Figure S5: Mass spectrum of DHMF; Figure S6: Mass spectrum of FA; Figure S7: Mass spectrum of MFA; Figure S8: Mass spectrum of FAM; Figure S9: Mass spectrum of MFAM; Figure S10: Mass spectrum of imine intermediates; Figure S11: Mass spectrum of HMTHFA; Figure S12: Mass spectrum of BATHMF; Figure S13: Mass spectrum of DHTHMF; Figure S14: Mass spectrum of OABCO; Figure S15: Mass spectrum of Schiff base intermediate; Figure S16: Mass spectrum of Schiff base hydrogenation product; Figure S17: Gas chromatogram of the HMF catalytic reaction over the Ni_1_Zn_4_Al_8_O_x_ catalyst. Reaction conditions: 1 g catalyst, 5 g HMF, 25 mL 1,4-Diox, 11.5 g NH_3_, 4.5 MPa H_2_, 90 °C for 6 h and then 210 °C for 18 h; Figure S18: Gas chromatogram of the HMF catalytic reaction over the Ni_4_Zn_4_Al_8_O_x_ catalyst. Reaction conditions: 5 g HMF, 25 mL 1,4-Diox, 11.5 g NH_3_, 4.5 MPa H_2_, 90 °C for 2 h; Figure S19: Gas chromatogram of the HMF catalytic reaction over the Ni_4_Zn_4_Al_8_O_x_ catalyst. Reaction conditions: 5 g HMF, 25 mL 1,4-Diox, 11.5 g NH_3_, 4.5 MPa H_2_, 90 °C for 4 h.

## Abbreviations

HMF	5-hydroxymethylfurfural
FAM	furfurylamine
FA	furoic acid
BAMF	2,5-bis(aminomethyl) furan
HMFA	5-hydroxymethylfurfurylamine
DHMF	2,5-dihydroxymethylfuran
MFA	5-methylfurfuryl alcohol
MFAM	5-methyl-2-furanamine
OABCO	8-oxa-3-azabicyclo [3.2.1]octane
BAMTHF	2,5-bis(aminomethyl)tetrahydrofuran
HMTHFA	5-hydroxymethyltetrahydrofurfurylamine
DHMTHF	2,5-dihydroxymethyl tetrahydrofuran
