# Supplementary material for: One-Pot Amination of 5-Hydroxymethylfurfural to 2,5-Bis(aminomethyl)furan over NiZnAl Catalysts
Source: Molecules. 2026 May 10;31(10):1600. doi: 10.3390/molecules31101600 (PMC13209339; doi:10.3390/molecules31101600)
Supplement: Supplementary file 1 [file molecules-31-01600-s001.zip › molecules-4253916-supplementary.pdf]

Supporting Information

One-pot amination of 5-hydroxymethylfurfural (HMF) to 2,5-bis(aminomethyl)furan (BAMF) over NiZnAl catalysts

Cong Wang, Xin Li*, Junqi Zhao*, Bin Sun, Xiaoxin Zhang, Xuhong Mu*

State Key Laboratory of Petroleum Molecular & Process Engineering, SINOPEC Research Institute of Petroleum Processing, Beijing 100083, China

Corresponding author

* Email address: lixin.ripp@sinopec.com; Tel.: +86-010-82368306.

zhaojq.ripp@sinopec.com; Tel.: +86-010-82368790.

muxuhong.ripp@sinopec.com; Tel.: +86-010-82368251.

Table S1. Reductive amination of HMF over $Ni_4Zn_4Al_8O_x$ catalyst.

Entry	Catalyst	T/°C	t/h	Imine intermediate/%	Schiff base intermediate/%	HMFA/%	BAMF/%
1	-	90	2	5.94	16.37	-	-
2	-	90	4	11.34	46.81	-	-
3	-	90	6	20.4	73.36	4.74	-
4	-	90	8	16.22	69.25	4.12	-
5	-	90	10	14.92	71.37	3.71	0.32
6	$Ni_4Zn_4Al_8O_x$	90	2	12.68	47.05	35.96	0.18
7	$Ni_4Zn_4Al_8O_x$	90	4	4.67	16.14	73.82	1.69
8	$Ni_4Zn_4Al_8O_x$	90	6	-	-	92.89	3.84
9	$Ni_4Zn_4Al_8O_x$	90	8	-	-	90.57	5.69
10	$Ni_4Zn_4Al_8O_x$	90	10	-	-	87.27	8.76
11	-	120	2	-	52.45	35.22	-
12	-	120	4	-	16.72	75.72	-
13	-	120	6	-	2.61	87.82	-
14	-	120	8	-	1.59	90.28	-
15	-	120	10	-	-	92.52	-
16	$Ni_4Zn_4Al_8O_x$	120	2	-	13.69	61.38	2.25
17	$Ni_4Zn_4Al_8O_x$	120	4	-	-	74.14	3.66
18	$Ni_4Zn_4Al_8O_x$	120	6	-	-	71.78	7.06
19	$Ni_4Zn_4Al_8O_x$	120	8	-	-	68.89	11.45
20	$Ni_4Zn_4Al_8O_x$	120	10	-	-	66.04	15.15

Reaction conditions: 5 g HMF, 25 mL 1,4-Diox, 11.5 g NH_3, 4.5 MPa H_2.

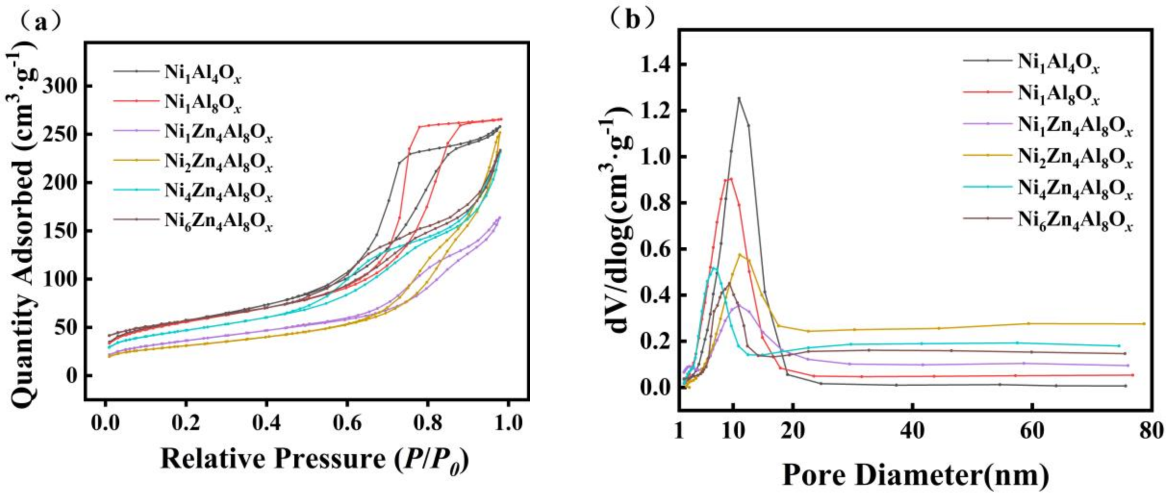

Figure S1. (a) N_2 adsorption–desorption isotherms and (b) Pore size distribution diagram of the prepared catalysts.

Table S2. Chemical property of the NiZnAl catalysts.

**Entry**	**Catalyst**	**H_2 adsorption capacity (mmol/g)[a]**	**Weak acid amount (μmol/g)[b]**	**Medium acid amount (μmol/g)[b]**	**Strong acid amount (μmol/g)[b]**	**Total acid amount (μmol/g)[b]**	**Ni 2p3/2 (eV)[c]**
1	$Ni_1Al_4O_x$	8.2	0.0133	0.0235	0.0302	0.067	—
2	$Ni_1Al_8O_x$	7.8	0.0184	0.0181	0.0245	0.061	—
3	$Ni_1Zn_4Al_8O_x$	4.3	0.0209	0.0111	—	0.032	852.70
4	$Ni_2Zn_4Al_8O_x$	5.1	0.0220	0.0120	—	0.034	852.75
5	$Ni_4Zn_4Al_8O_x$	5.6	0.0183	0.0187	—	0.037	852.98
6	$Ni_6Zn_4Al_8O_x$	6.5	0.0100	0.0314	0.0036	0.045	853.36

[a] H_2 consumption obtained after quantitative analysis of the reduction peaks from H_2-TPD.

[b] Acid amount obtained after quantitative analysis of the reduction peaks from NH_3-TPD.

[c] Binding Energy of Ni $2p_{3/2}$ in XPS

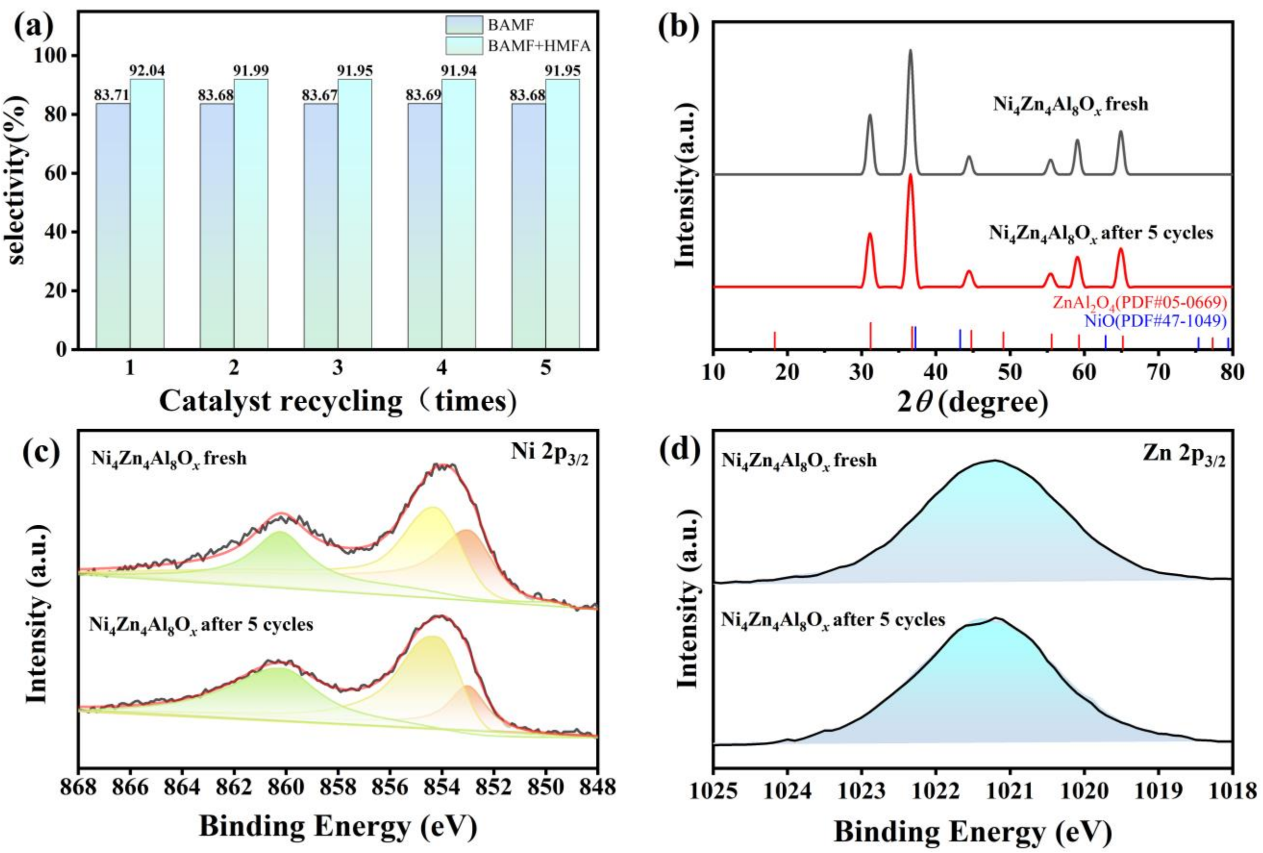

Figure S2. (a)Effect of recycling times on the catalytic performance of the $Ni_4Zn_4Al_8O_x$ catalyst.

(b) XRD patterns of fresh and 5 times recycled $Ni_4Zn_4Al_8O_x$ catalyst.

(c) Ni $2p_{3/2}$ XPS spectra of fresh and 5 times recycled $Ni_4Zn_4Al_8O_x$ catalyst.

(d) Zn $2p_{3/2}$ XPS spectra of fresh and 5 times recycled $Ni_4Zn_4Al_8O_x$ catalyst.

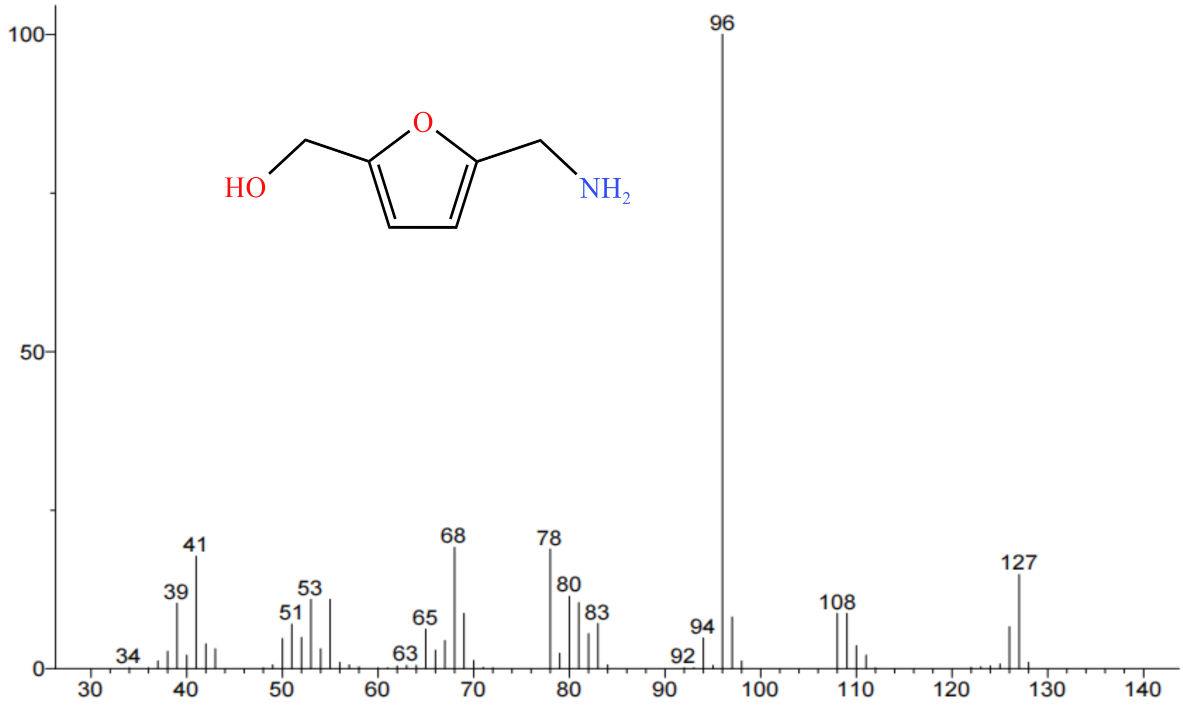

Figure S3. Mass spectrum of HMFA.

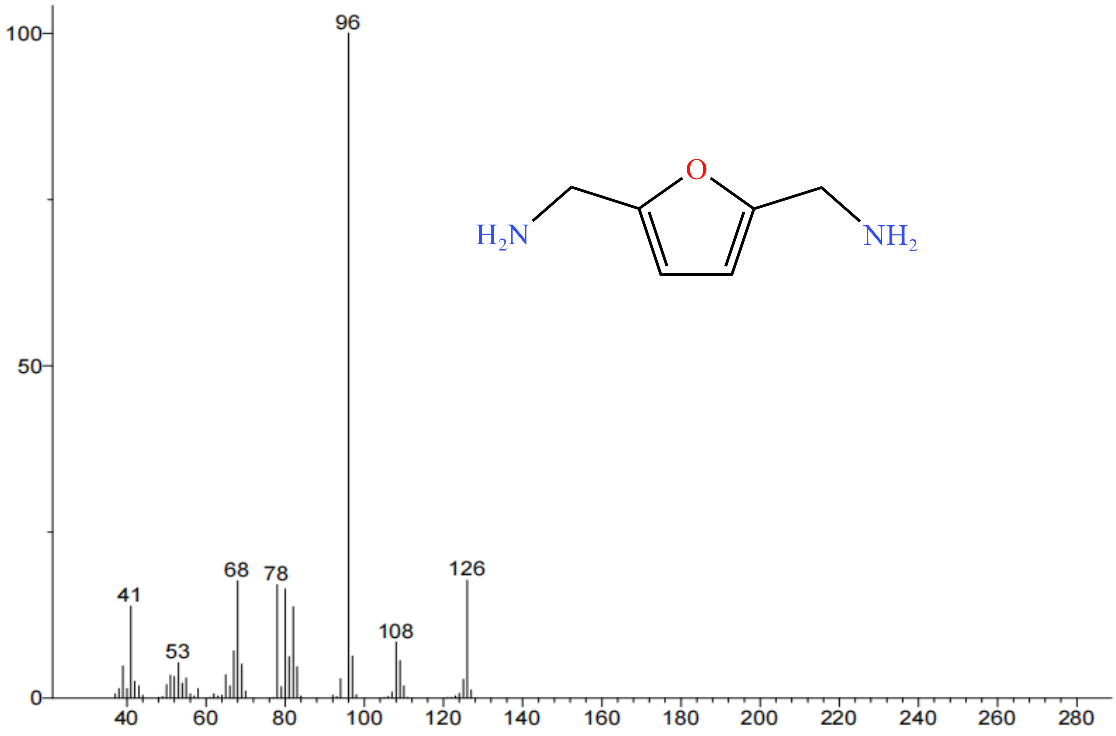

Figure S4. Mass spectrum of BAMF.

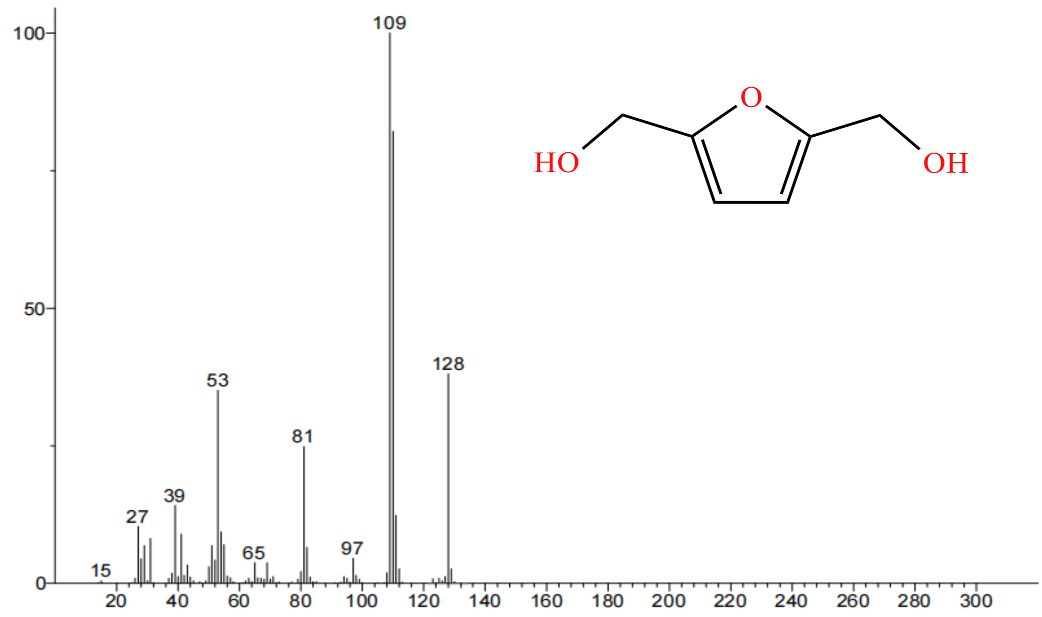

Figure S5. Mass spectrum of DHMF.

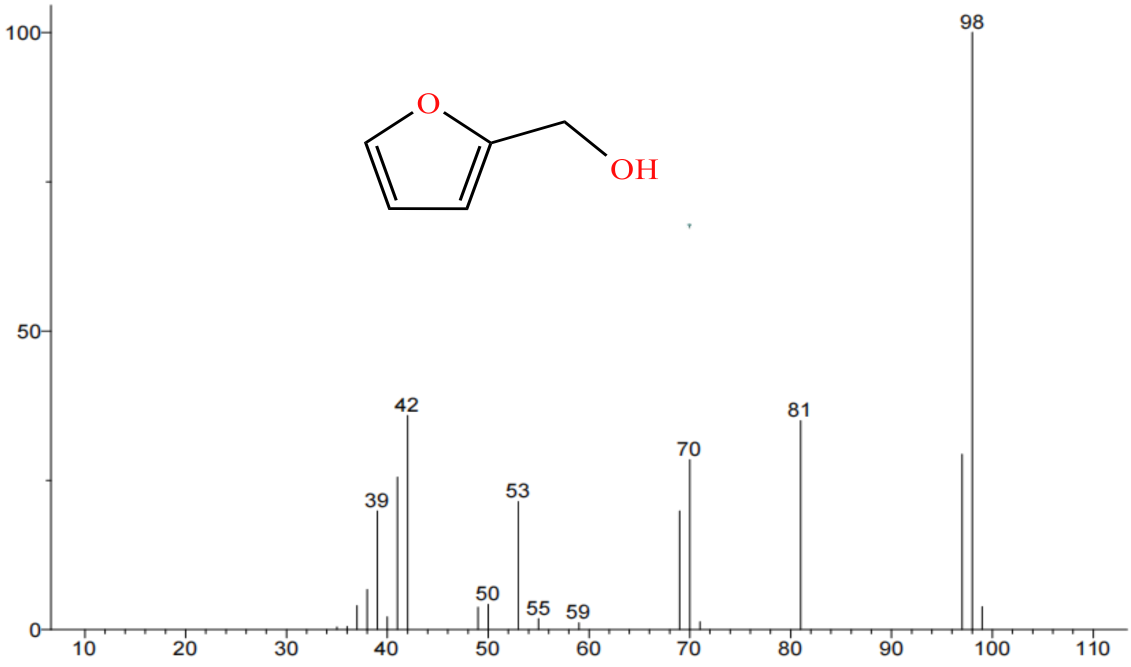

Figure S6. Mass spectrum of FA.

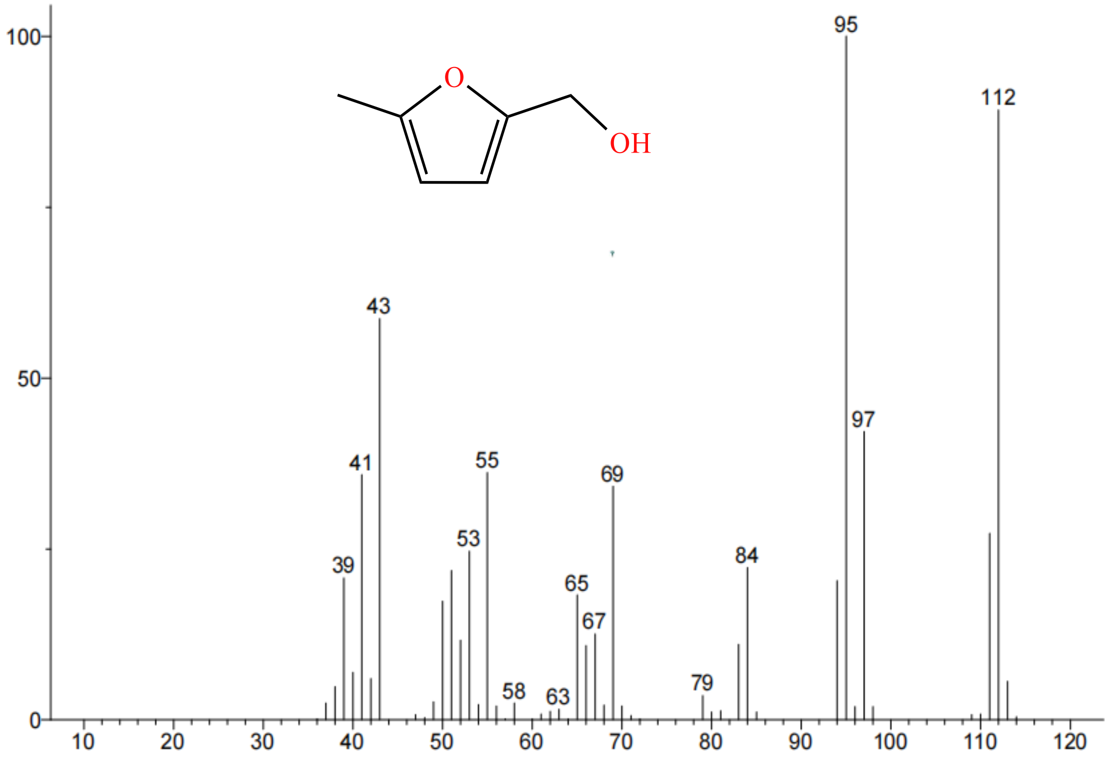

Figure S7. Mass spectrum of MFA.

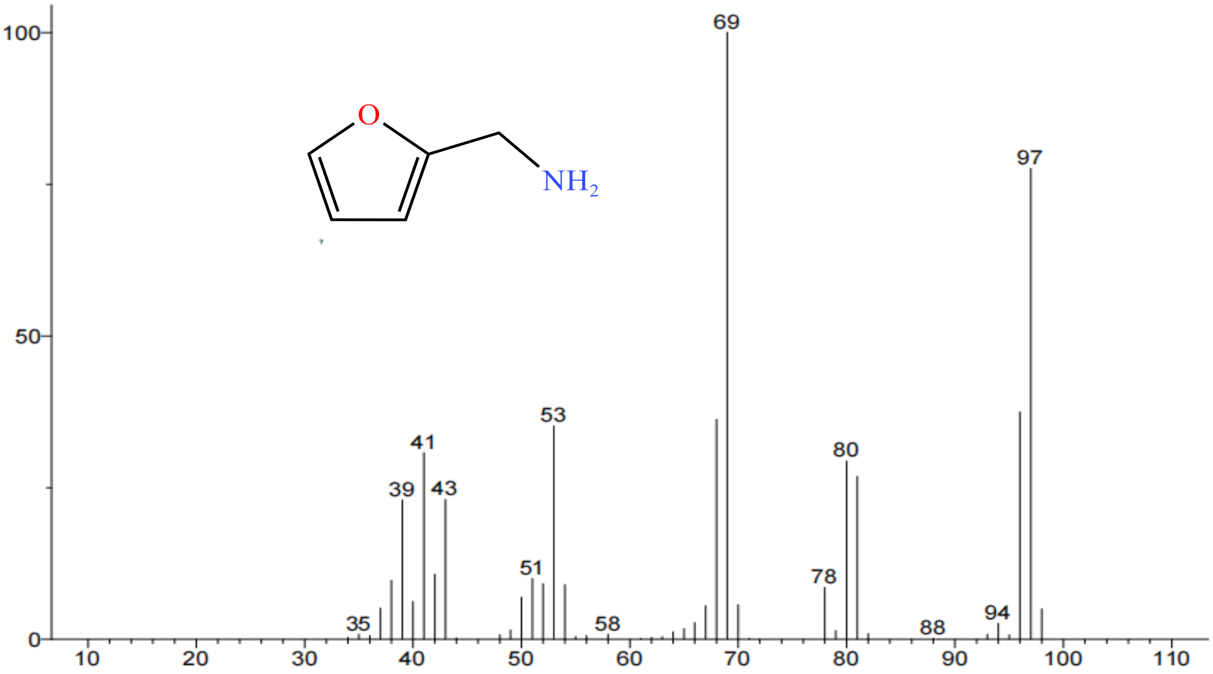

Figure S8. Mass spectrum of FAM.

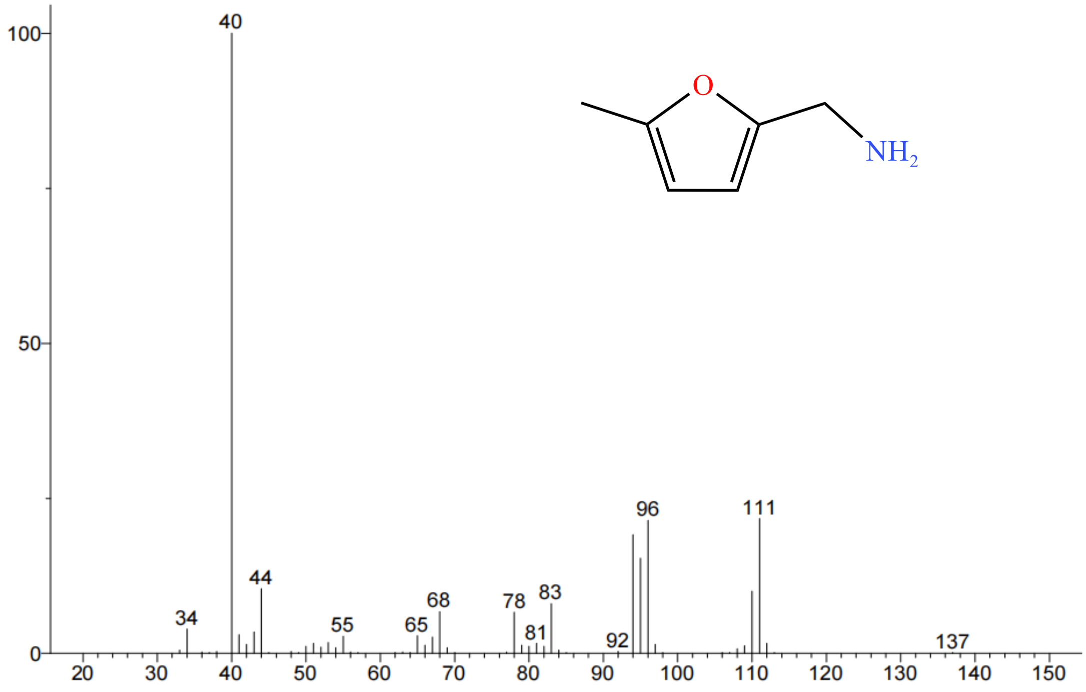

Figure S9. Mass spectrum of MFAM.

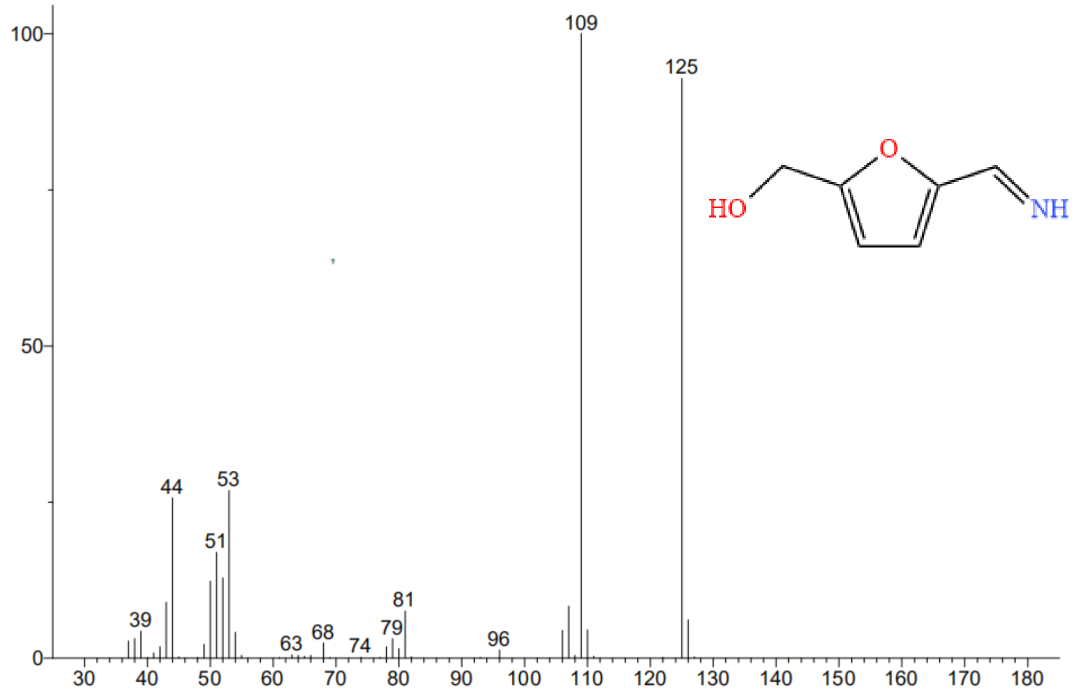

Figure S10. Mass spectrum of imine intermediates.

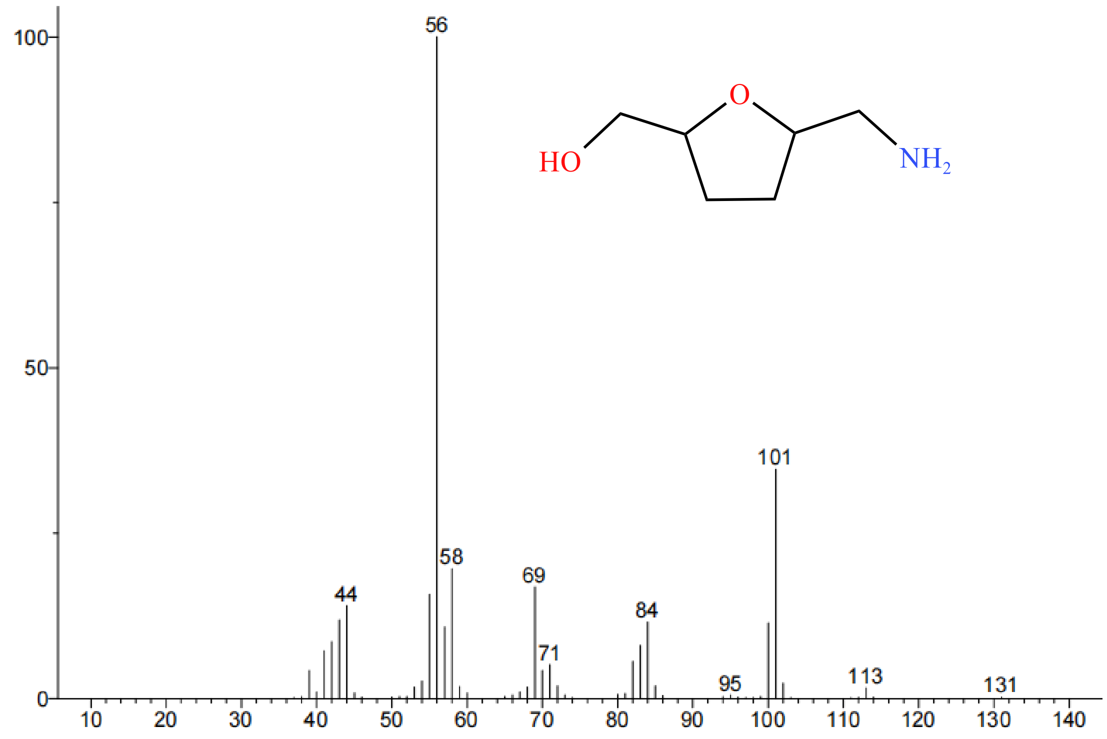

Figure S11. Mass spectrum of HMTHFA.

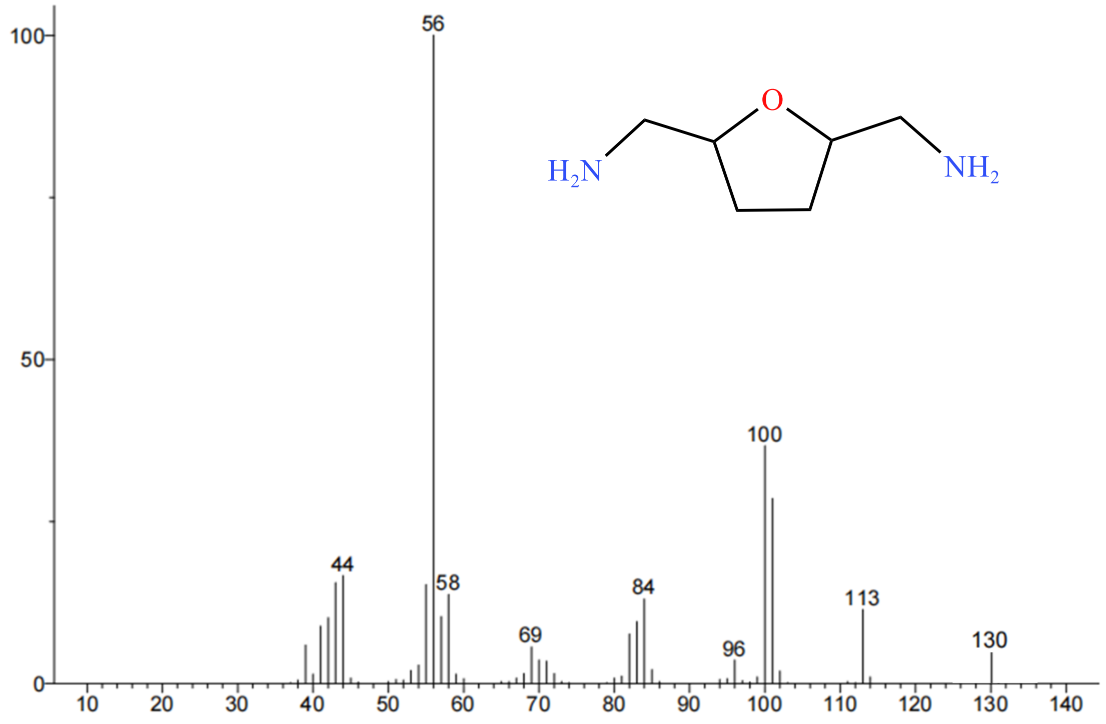

Figure S12. Mass spectrum of BATHMF.

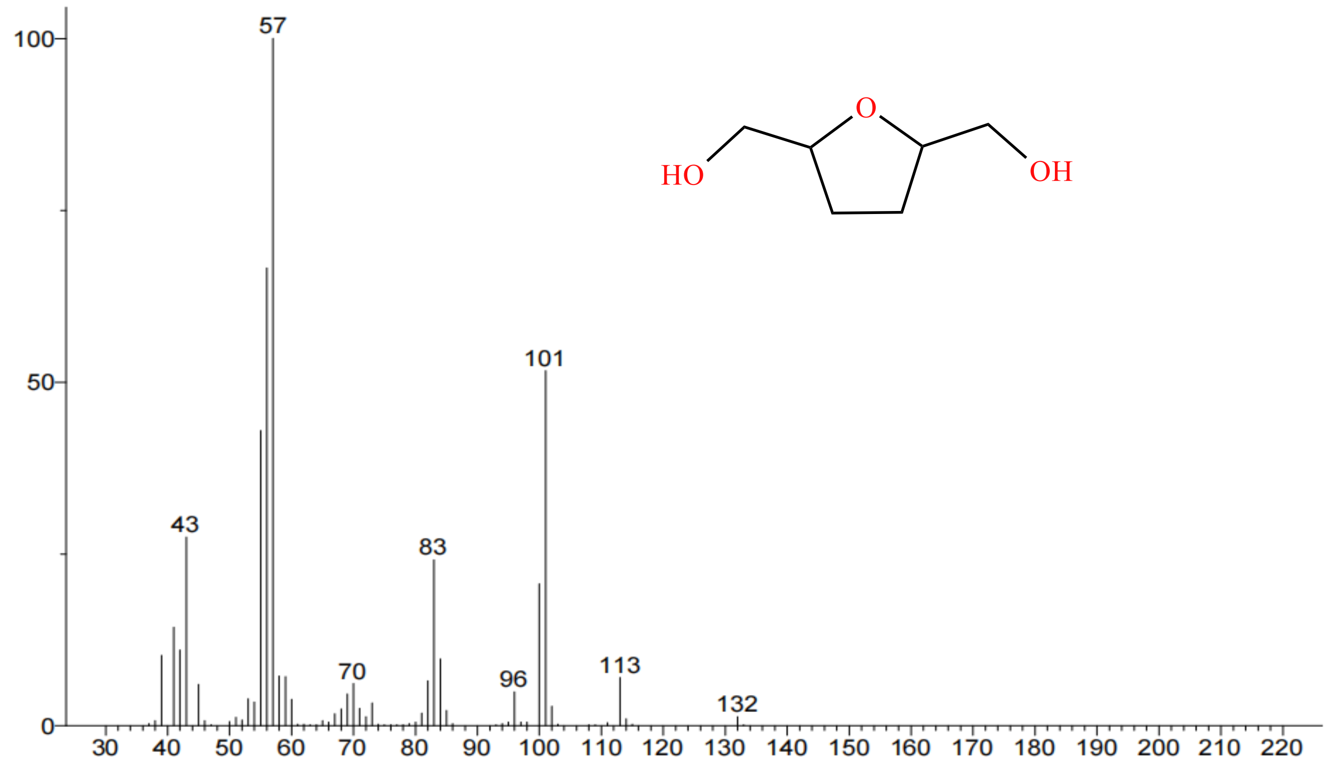

Figure S13. Mass spectrum of DHTHMF.

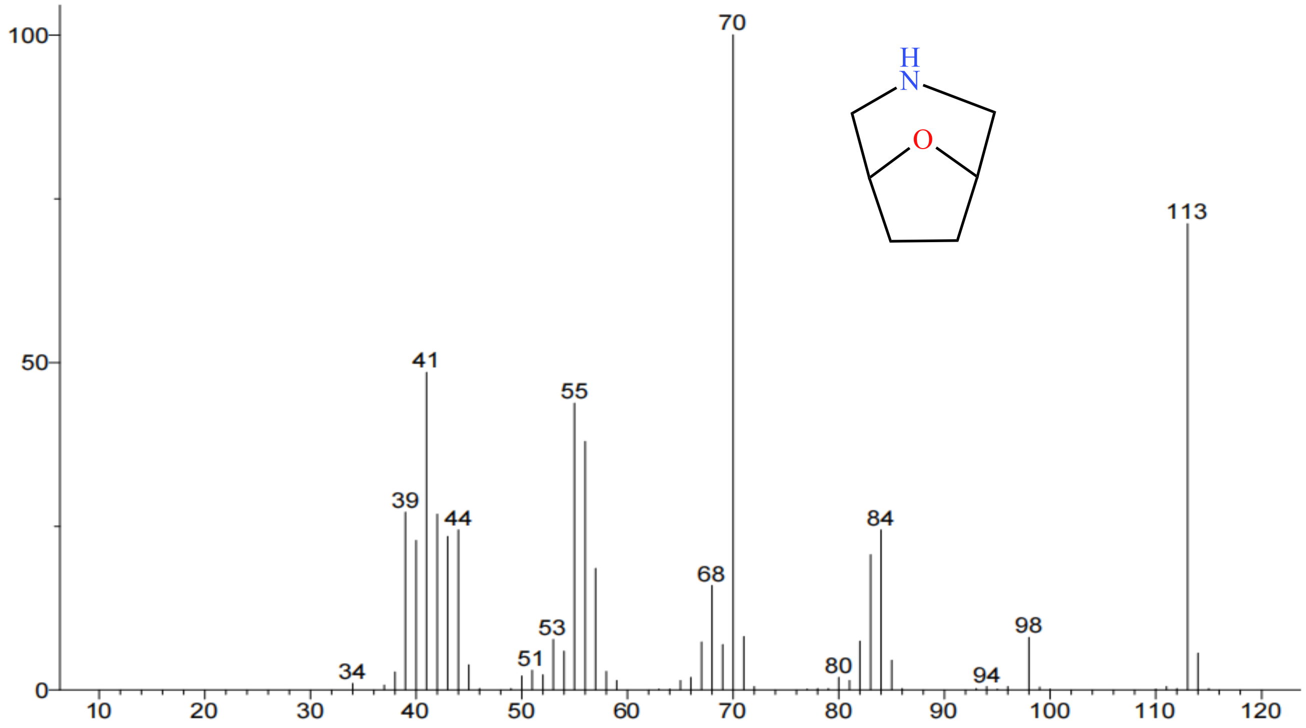

Figure S14. Mass spectrum of OABCO.

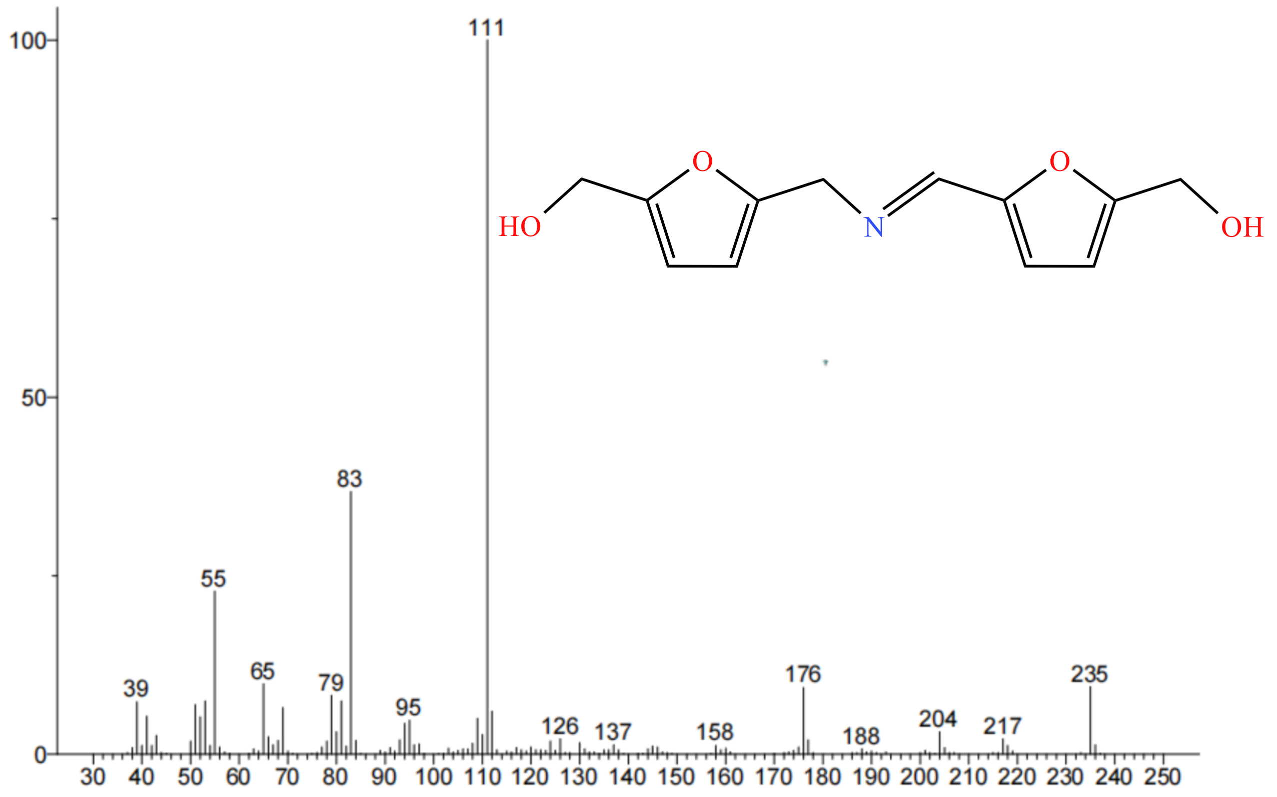

Figure S15. Mass spectrum of Schiff base intermediate.

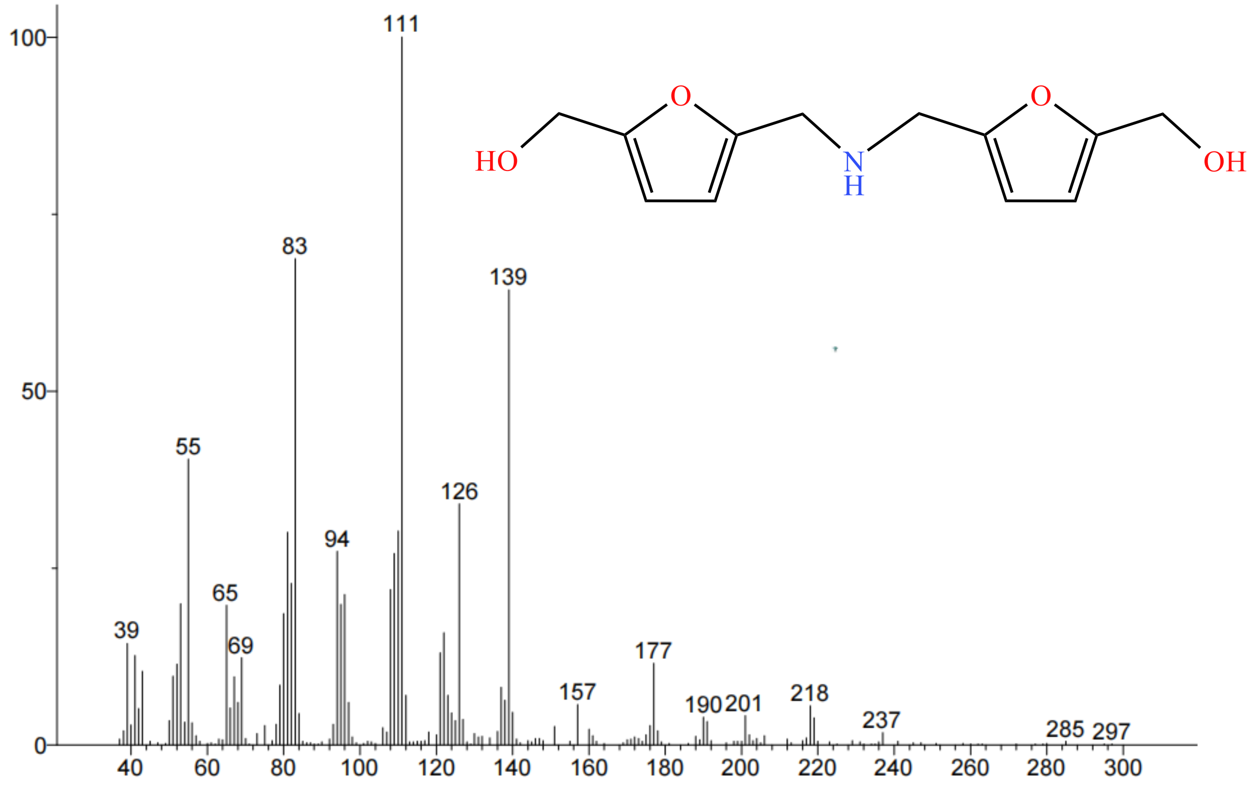

Figure S16. Mass spectrum of Schiff base hydrogenation product.

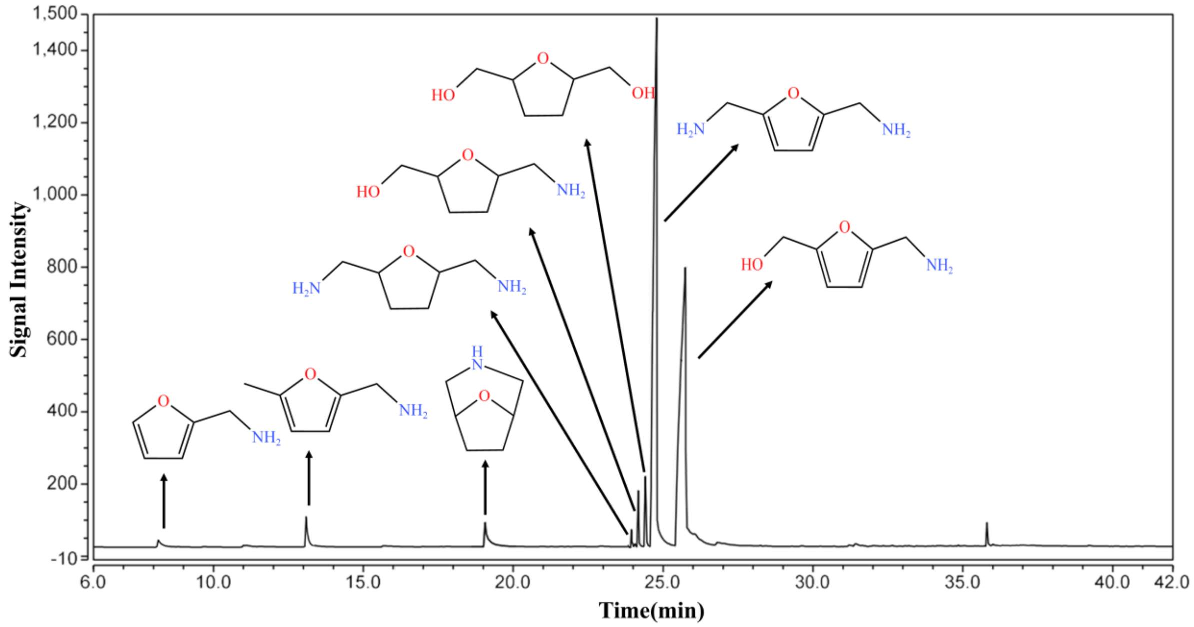

Figure S17. Gas chromatogram of the HMF catalytic reaction over the $Ni_1Zn_4Al_8O_x$ catalyst. Reaction conditions: 1 g catalyst, 5 g HMF, 25 mL 1,4-Diox, 11.5 g NH_3, 4.5 MPa H_2, 90 °C for 6 h and then 210 °C for 18 h.

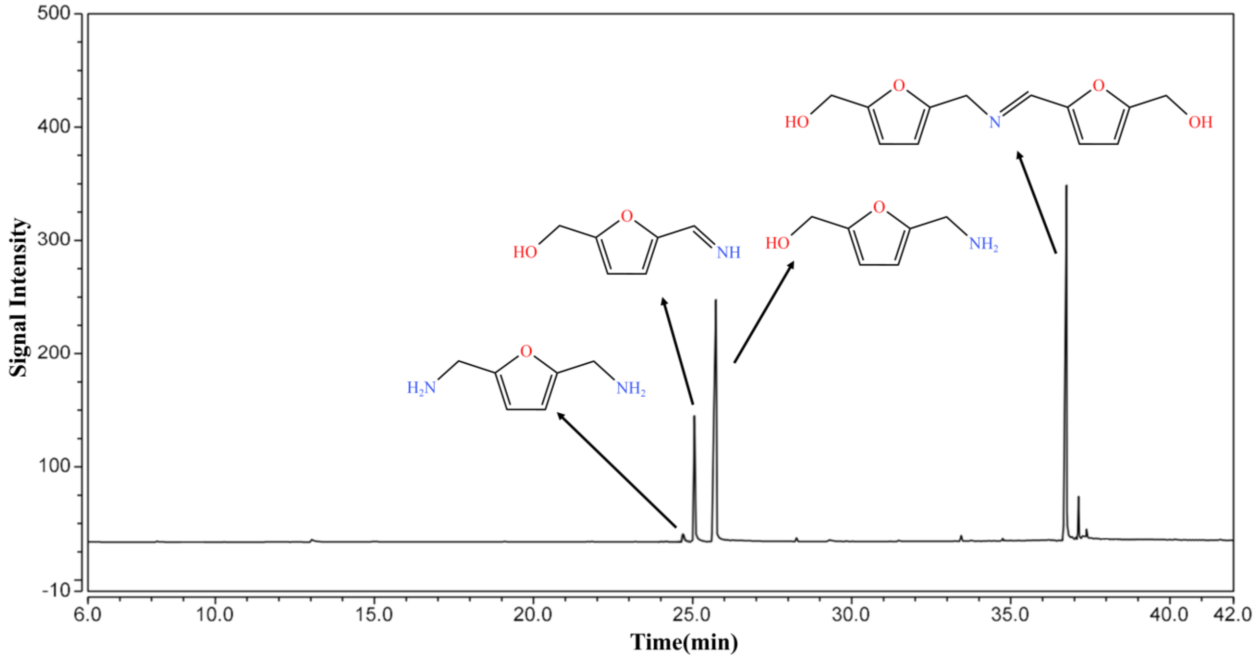

Figure S18. Gas chromatogram of the HMF catalytic reaction over the $Ni_4Zn_4Al_8O_x$ catalyst. Reaction conditions: 5 g HMF, 25 mL 1,4-Diox, 11.5 g NH_3, 4.5 MPa H_2, 90 °C for 2 h.

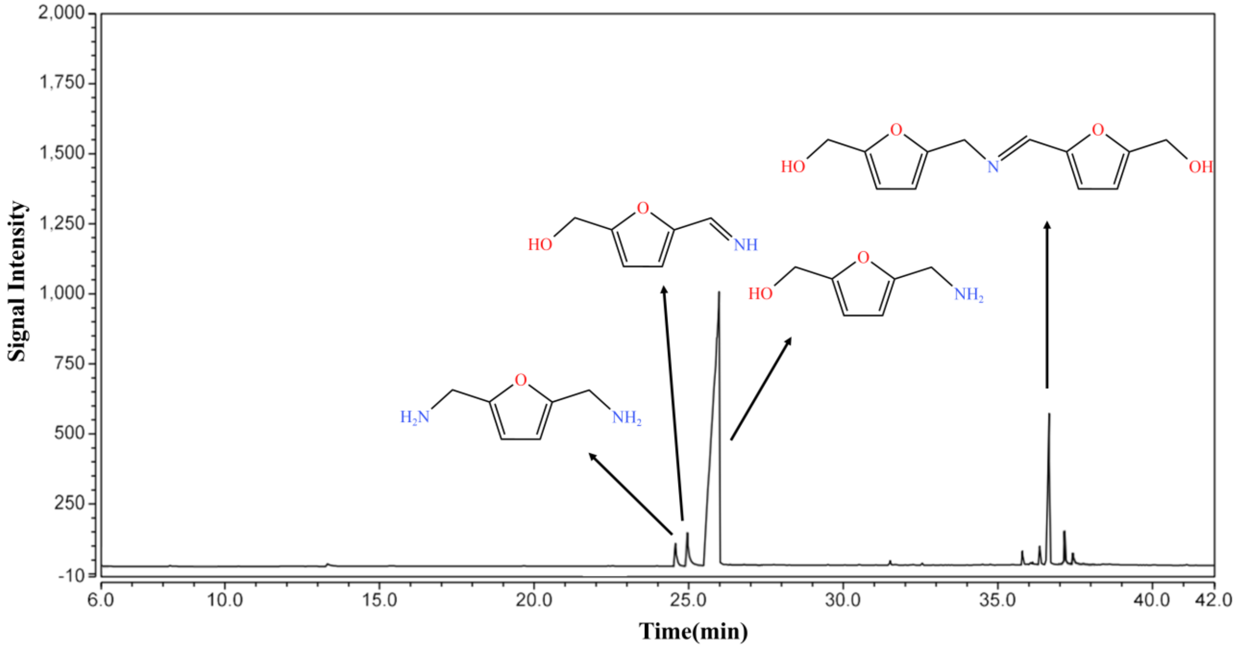

Figure S19. Gas chromatogram of the HMF catalytic reaction over the $Ni_4Zn_4Al_8O_x$ catalyst.

Reaction conditions: 5 g HMF, 25 mL 1,4-Diox, 11.5 g NH_3, 4.5 MPa H_2, 90 °C for 4 h.
